# Identification of glyoxalase A in group B *Streptococcus* and its contribution to methylglyoxal tolerance and virulence

**DOI:** 10.1128/iai.00540-24

**Published:** 2025-02-26

**Authors:** Madeline S. Akbari, Luke R. Joyce, Brady L. Spencer, Amanda Brady, Kevin S. McIver, Kelly S. Doran

**Affiliations:** 1 Department of Immunology and Microbiology, School of Medicine, University of Colorado Anschutz Medical Campus 12225 , Aurora, Colorado, USA; 2 Cell Biology and Molecular Genetics, Maryland Pathogen Research Institute, University of Maryland 171289 , College Park, Maryland, USA; University of Illinois Chicago, Chicago, Illinois, USA

**Keywords:** group B Streptococcus, *Streptococcus agalactiae*, host-pathogen interactions, glycolysis, glyoxalase, methylglyoxal, bacteremia, neutrophils

## Abstract

Group B *Streptococcus* (GBS) is a Gram-positive pathobiont that commonly colonizes the gastrointestinal and lower female genital tracts but can cause sepsis and pneumonia in newborns and is a leading cause of neonatal meningitis. Despite the resulting disease severity, the pathogenesis of GBS is not completely understood, especially during the early phases of infection. To investigate GBS factors necessary for bloodstream survival, we performed a transposon (Tn) mutant screen in our bacteremia infection model using a GBS *mariner* transposon mutant library previously developed by our group. We identified significantly underrepresented mutations in 623 genes that contribute to survival in the blood, including those encoding known virulence factors such as capsule, the β-hemolysin, and inorganic metal ion transport systems. Most of the underrepresented genes have not been previously characterized or studied in GBS, including *gloA* and *gloB,* which are homologs for genes involved in methylglyoxal (MG) detoxification. MG is a byproduct of glycolysis and a highly reactive toxic aldehyde that is elevated in immune cells during infection. Here, we observed MG sensitivity across multiple GBS isolates and confirmed that *gloA* contributes to MG tolerance and invasive GBS infection. We show specifically that *gloA* contributes to GBS survival in the presence of neutrophils and depleting neutrophils in mice abrogates the decreased survival and infection of the *gloA* mutant. The requirement of the glyoxalase pathway during GBS infection suggests that MG detoxification is important for bacterial survival during host-pathogen interactions.

**IMPORTANCE:**

A transposon-mutant screen of group B *Streptococcus* (GBS) in a bacteremia mouse model of infection revealed virulence factors known to be important for GBS survival such as the capsule, β-hemolysin/cytolysin, and genes involved in metal homeostasis. Many uncharacterized factors were also identified including genes that are part of the metabolic pathway that breaks down methylglyoxal (MG). The glyoxalase pathway is the most ubiquitous metabolic pathway for MG breakdown and is only a two-step process using glyoxalase A (*gloA*) and B (*gloB*) enzymes. MG is a highly reactive byproduct of glycolysis and is made by most cells. Here, we show that in GBS, the first enzyme in the glyoxalase pathway, encoded by *gloA*, contributes to MG resistance and blood survival. We further demonstrate that GloA contributes to GBS survival against neutrophils *in vitro* and *in vivo* and, therefore, is an important virulence factor required for invasive infection.

## INTRODUCTION

*Streptococcus agalactiae* (group B *Streptococcus*, GBS) is an opportunistic pathogen that commonly resides in the gastrointestinal and lower female genital tracts but can cause infection in newborns and is also increasingly associated with non-pregnant individuals, especially older adults and patients with diabetes (1–3). GBS asymptomatically colonizes the vaginal tract in up to 30% of people but can instigate complications during pregnancy and birth, such as preterm labor, and serious infections in newborns, such as sepsis, pneumonia, and meningitis (1, 4–6). Research into GBS intrauterine infection during pregnancy thus far indicates that GBS-activated inflammatory pathways ultimately result in preterm births (7). If GBS is vertically transferred to the neonate, the resulting infection is categorized as either early-onset disease (EOD, 0–6 days of life) or late-onset disease (LOD, 7–90 days of life) depending on the timing of symptom presentation (8). Neonatal meningitis caused by both EOD and LOD GBS disease requires a sustained level of bacteremia prior to the penetration into the brain and, even after treatment, frequently results in long-lasting neurological effects and long-term morbidity (4, 5). Although intrapartum antibiotic prophylaxis is administered to colonized pregnant women to prevent the detrimental effects of GBS infection, GBS isolates are increasing in resistance to second-line antibiotics over time (9), and intrapartum antibiotic prophylaxis is not effective in preventing LOD. Therefore, studying the GBS pathogenesis of meningitis, including bacteremia, is important for the development of novel treatments and therapeutics to prevent GBS infection and reduce morbidity and mortality.

Previous work has determined the GBS transcriptome as well as genes necessary for survival in human blood *in vitro* and for survival of the murine female reproductive tract (10–13). These data sets as well as other studies have shown that GBS possesses an arsenal of virulence factors that directly contribute to pathogenesis such as β-hemolysin/cytolysin, superoxide dismutase, capsule, adherence proteins, and metal transport systems (10, 14, 15). β-hemolysin/cytolysin (βH/C) and capsular polysaccharides are the most well-studied factors associated with GBS pathogenesis and are regulated by the well-known two-component system, CovR/S (15). βH/C is an ornithine rhamnolipid pigment synthesized by the *cyl* operon and has both hemolytic and cytolytic capabilities against a variety of host cells including red blood cells, neutrophils, macrophages, and epithelial cells (16–19). As a result, βH/C has been shown to contribute to GBS blood, lung, and brain infection (17, 20). Capsular polysaccharide is surface-associated and made up of different arrangements of monosaccharides that form capsular repeat units (21, 22). There are 10 known GBS capsular serotypes with serotype III being highly associated with neonatal infections, such as meningitis, and which is overrepresented in invasive isolates worldwide (9, 23, 24). Group B streptococcal capsular polysaccharide was first studied over 40 years ago and has been shown to help GBS evade host immune defenses by mimicking host antigens and blocking complement-mediated opsonophagocytic killing as well as to facilitate GBS biofilm formation (22, 25–27). Despite these studies, the contribution of GBS metabolism to colonization and infection *in vivo* has been a neglected area of study in the field (10, 15).

Here, we performed a transposon-mutant screen (Tn-sequencing) using a murine bacteremia model to discover additional genes necessary for GBS fitness in murine blood *in vivo*. GBS survival within the blood is an essential prerequisite to penetrating the blood-brain barrier and subsequent development of meningitis. Tn-sequencing allows for the identification of genes that may be continuously expressed but are essential in certain environments. Here, we identify that the glyoxalase pathway is required for GBS bloodstream survival. The glyoxalase pathway consists of two genes, *gloA* and *gloB,* and is involved in methylglyoxal detoxification (28, 29). Methylglyoxal (MG) is a toxic byproduct of normal cell metabolism (30), and we confirm that the first enzyme in the pathway, encoded by *gloA,* contributes to GBS MG detoxification and invasive infection. Furthermore, we found that *gloA* is necessary for GBS neutrophil survival, and depleting neutrophils rescue the *gloA* mutant *in vivo*.

## RESULTS

### Genome-wide analysis of GBS factors involved in bloodstream survival

**Fig 1:**
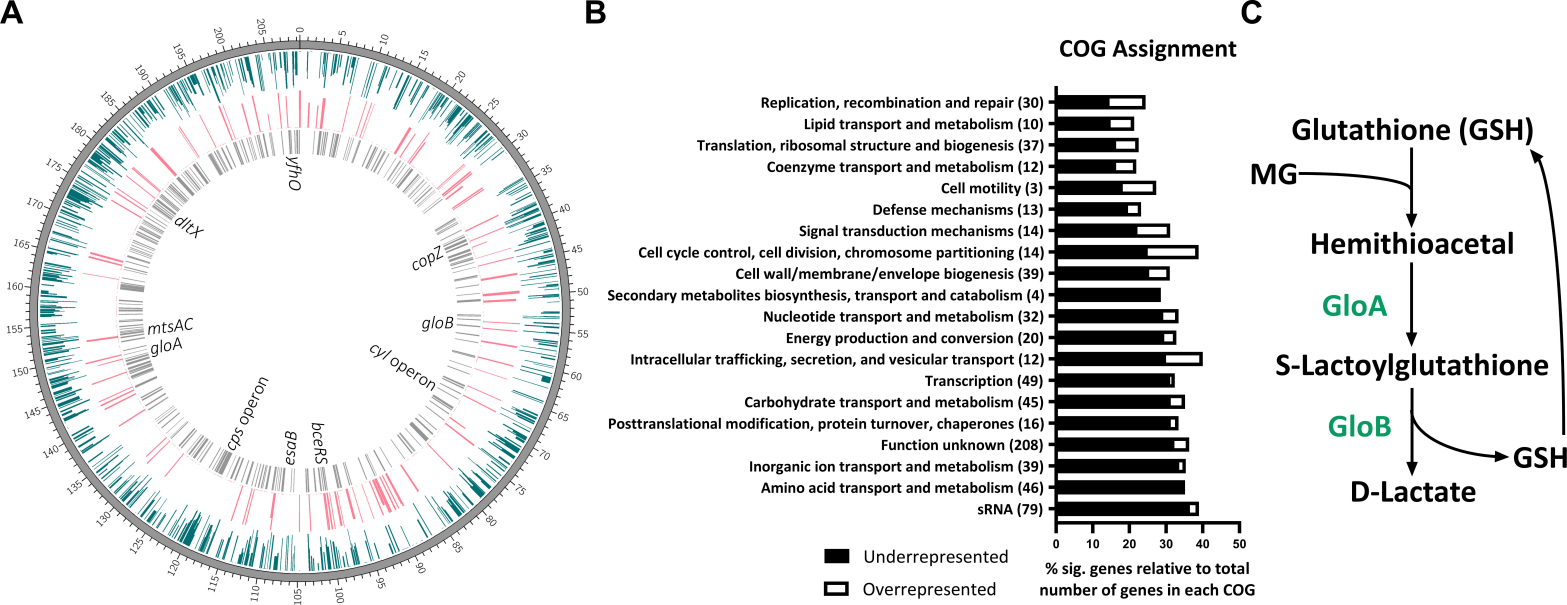
*In vivo* transposon mutant sequencing of GBS survival in the blood. (**A**) CIRCOS atlas representation of the GBS CJB111 genome is shown with a base pair ruler on the outer ring. The inner ring in blue shows log_2_FC (0 to −5/max) of significantly underrepresented genes (*P*_adj_ < 0.05). The next inner ring in pink shows log_2_FC (0 to 5/max) of significantly overrepresented genes (*P*_adj_ < 0.05). The most inner ring in gray denotes genes with *P*_adj_ < 0.001. Underrepresented genes or operons of interest are labeled in the center (also listed in Table 1). (**B**) COG assignments for significant gene hits normalized to the total number of GBS genes in each COG. The total number of significant genes in each COG is in parentheses. (**C**) Diagram of the glyoxalase pathway for MG breakdown. Significance determined by (A and B) TRANSIT analysis and Trimmed Total Reads normalization with *P*_adj_ < 0.05 and log_2_FC < −2 or >2.

To identify genes necessary for GBS survival in murine blood, we utilized a bacteremia model of infection with our previously described Tn mutant library in the CJB111 strain, a GBS isolate from a case of neonatal bacteremia without focus (17, 31–33). Briefly, mice were intravenously infected with the Tn mutant library and the infection was allowed to progress up to 27 h. The input Tn mutant library and libraries recovered from the blood were processed as described in Materials and Methods. To identify transposon insertion sites, sequenced reads were mapped to the GBS CJB111 genome, which identified 623 genes as significantly underrepresented (*P*_adj_ < 0.05, log_2_FC < −1) and 95 genes as significantly overrepresented (*P*_adj_ < 0.05, log_2_FC > 1) in the blood compared to the input library (Fig. 1A) (Table S1). The significant gene hits were equally distributed across the genome. Significant genes were then assigned clusters of orthologous groups of proteins (COGs). The number of significant gene hits in each COG was normalized to the total number of genes in each COG revealing sRNA, amino acid transport and metabolism, and inorganic ion transport and metabolism as the COGs containing the most underrepresented genes during GBS survival in the blood (Fig. 1B). We detected many classes of GBS virulence factors and genes known to contribute to GBS infection as significantly underrepresented (Table 1). Some of these genes are involved in hemolytic pigment biosynthesis, capsule biosynthesis, two-component regulatory systems, metal transport, glutamine transport, and purine metabolism. When we investigated other underrepresented genes that have not been previously characterized in GBS, we found homologous genes to glyoxalase A and B of the glyoxalase pathway to both be significantly underrepresented with fold changes of −18.38 and −25.63, respectively (Table 1). The glyoxalase pathway is a ubiquitous two-step process found across all kingdoms of life and is the primary mechanism of MG breakdown (Fig. 1C) (28). A highly reactive carbonyl byproduct of normal cell metabolism, most MG is primarily generated from glycolysis, but can also be produced from other metabolic pathways such as lipid and protein metabolism (34).

**TABLE 1:** Important GBS virulence factors contribute to survival in blood^*a*^

Locus tag	Gene name	Description	Fold change	Adj. *P*-value	Function	GBS reference
Virulence factors						
ID870_00715	*dltX*	Teichoic acid D-Ala incorporation-associated protein DltX	−21.26	0	Lipoteichoic acid biosynthesis	(35)
ID870_01745	*scpB*	Segregation/condensation protein B	−27.67	0	Complement evasion and adhesion	(36)
ID870_02600	*pil2a-bp*	PI-2a subunit	−8.22	0	Adhesion	(37)
ID870_04170	*esxA1*	WXG100 family type VII secretion target	−4.99	0.00432	Type VII Secretion System	(12)
ID870_04190	*esaB*	EsaB/YukD family protein	−20.68	0.02043	Type VII Secretion System	
ID870_05725	*iagA*	Glycosyltransferase	−20.97	0	Lipid biosynthesis	(38)
ID870_05905	*cylK*	CylK protein	−7.11	0.00118	β-hemolysin biosynthesis	(39, 40)
ID870_05910	*cylJ*	CylJ protein	−2.73	0.03089	β-hemolysin biosynthesis	
ID870_05915	*cylH/I*	Beta-ketoacyl-[acyl-carrier-protein] synthase family protein	−4.23	0.0031	β-hemolysin biosynthesis	
ID870_05920	*cylF*	Aminomethyltransferase family protein	−12.91	0	β-hemolysin biosynthesis	
ID870_06030	*pil1-bp*	PI-1 major pilin	−7.01	0	Adhesion	(41)
ID870_07490	*brpA*	LCP family protein	−4.35	0	Biofilm regulation	(42)
ID870_09735	*yfhO*	YfhO family protein	−9.38	0.00063	Glycan biosynthesis	(43)
Capsule						
ID870_02780	*capA*	CapA family protein	−5.24	0.0017	Capsule biosynthesis	(22, 44, 45)
ID870_03485	*cps4A*	LCP family protein	−6.15	0	Capsule regulation	
ID870_03500	*cpsD*	Tyrosine-protein kinase	−7.36	0	Capsule biosynthesis	
ID870_03505	*cpsE*	Sugar transferase	−9.85	0	Capsule biosynthesis	
ID870_03510	*cpsF*	UDP-N-acetylglucosamine–LPS N-acetylglucosamine transferase	−26.72	0	Capsule biosynthesis	
ID870_03515	*cpsG*	Multidrug MFS transporter	−28.64	0	Capsule biosynthesis	
ID870_03530	*cpsN*	Glycosyltransferase family 2 protein	−11.31	0	Capsule biosynthesis	
ID870_03535	*cpsO*	Glycosyltransferase family 2 protein	−7.41	0	Capsule biosynthesis	
ID870_03540	*cpsJ*	Glycosyltransferase family 2 protein	−13.36	0	Capsule biosynthesis	
ID870_03545	*cpsK*	Glycosyltransferase family 52 protein	−28.25	0	Capsule biosynthesis	
ID870_03550	*cpsL*	Oligosaccharide flippase family protein	−22.16	0	Capsule biosynthesis	
ID870_03555	*neuB*	N-acetylneuraminate synthase	−5.21	0	Capsule biosynthesis	
ID870_03560	*neuC*	UDP-N-acetylglucosamine 2-epimerase (hydrolyzing)	−10.06	0	Capsule biosynthesis	
ID870_03565	*neuD*	Acetyltransferase	−19.97	0	Capsule biosynthesis	
Metal ion transport						
ID870_00250	*lmb*	Zinc ABC transporter substrate-binding protein	−7.36	0.01419	Zinc uptake and adhesion	(46)
ID870_02010	*mtsA*	Metal ABC transporter substrate-binding protein	−58.89	0	Manganese uptake	(32)
ID870_02020	*mtsC*	Metal ABC transporter permease	−7.31	0	Manganese uptake	
ID870_02070	*nikA*	Nickel ABC transporter or nickel/metallophore periplasmic binding protein	−10.85	0	Nickel/copper uptake (putative)	(32, 47)
ID870_02660	*fhuG*	Iron ABC transporter permease	−6.41	0	Siderophore-dependent iron uptake	(48)
ID870_02665	*fhuB*	Iron ABC transporter permease	−6.50	0	Siderophore-dependent iron uptake	
ID870_05550	*mntH*	Nramp family divalent metal transporter	−2.87	0.03162	Manganese uptake	(49)
ID870_07395	*copZ*	Heavy-metal-associated domain-containing protein	−68.12	0.00559	Copper efflux	(50)
ID870_08645	*adcC*	Metal ABC transporter ATP-binding protein	−32.67	0	Zinc uptake	(46)
Two-component systems (TCS)						
ID870_00330	*maeR*	Response regulator	−6.96	0	Malic acid metabolism regulation (TCS-15)	(51–53)
ID870_00705	*dltR*	Response regulator transcription factor	−6.02	0	Lipoteichoic acid regulation (TCS-14)	(35, 51, 52, 54)
ID870_04460	*ciaH*	HAMP domain-containing histidine kinase	−8.63	0	Antimicrobial peptide resistance (TCS-10)	(51, 52, 55)
ID870_04495	*bceS/nsrK*	Sensor histidine kinase	−10.41	0.0017	Antimicrobial resistance (TCS-9)	(51, 52, 56–58)
ID870_04500	*bceR/nsrR*	Response regulator transcription factor	−20.11	0.00118	Antimicrobial resistance (TCS-9)	
ID870_10420	*cssS*	HAMP domain-containing histidine kinase	−9.13	0	Two-component system (TCS-19)	(51, 52)
Metabolism						
ID870_00585	*purA*	Adenylosuccinate synthase	−15.03	0	Purine metabolism	(59)
ID870_02260	***gloA***	Lactoylglutathione lyase	−18.38	0	Methylglyoxal detoxification	
ID870_02270	*yvgN*	Aldo/keto reductase	−4.00	0.00518	Aldehyde detoxification	
ID870_02315	*glnQ*	Amino acid ABC transporter ATP-binding protein	−5.28	0.00063	Glutamine transport	(60)
ID870_02320	*glnP*	ABC transporter substrate-binding protein/permease	−4.69	0	Glutamine transport	
ID870_04545	*guaA*	Glutamine-hydrolyzing GMP synthase	−12.55	0	Purine metabolism	(59, 61)
ID870_06660	***gloB***	MBL fold metallo-hydrolase	−25.63	0.04532	Methylglyoxal detoxification	
ID870_08815	*argH*	Argininosuccinate lyase	−4.79	0.00063	Arginine metabolism	(13, 62, 63)
ID870_09305	*purB*	Adenylosuccinate lyase	−16.22	0	Purine metabolism	(59)
ID870_09420	*purC*	Phosphoribosylaminoimidazolesuccinocarboxamide synthase	−27.67	0.00118	Purine metabolism	(59)
ID870_09800	*guaB*	IMP dehydrogenase	−38.05	0	Purine metabolism	(59, 61)
ID870_10075	*argF*	Ornithine carbamoyltransferase	−13.36	0	Arginine metabolism	(13, 62, 63)
ID870_10080	*arcC*	Carbamate kinase	−9.58	0.00264	Arginine metabolism	

^*a*^ *P* value < 0.0001 is assigned a *P* value of 0 by TRANSIT analysis. Bold text indicates genes of interest.

### Methylglyoxal tolerance differs across GBS strains

**Fig 2:**
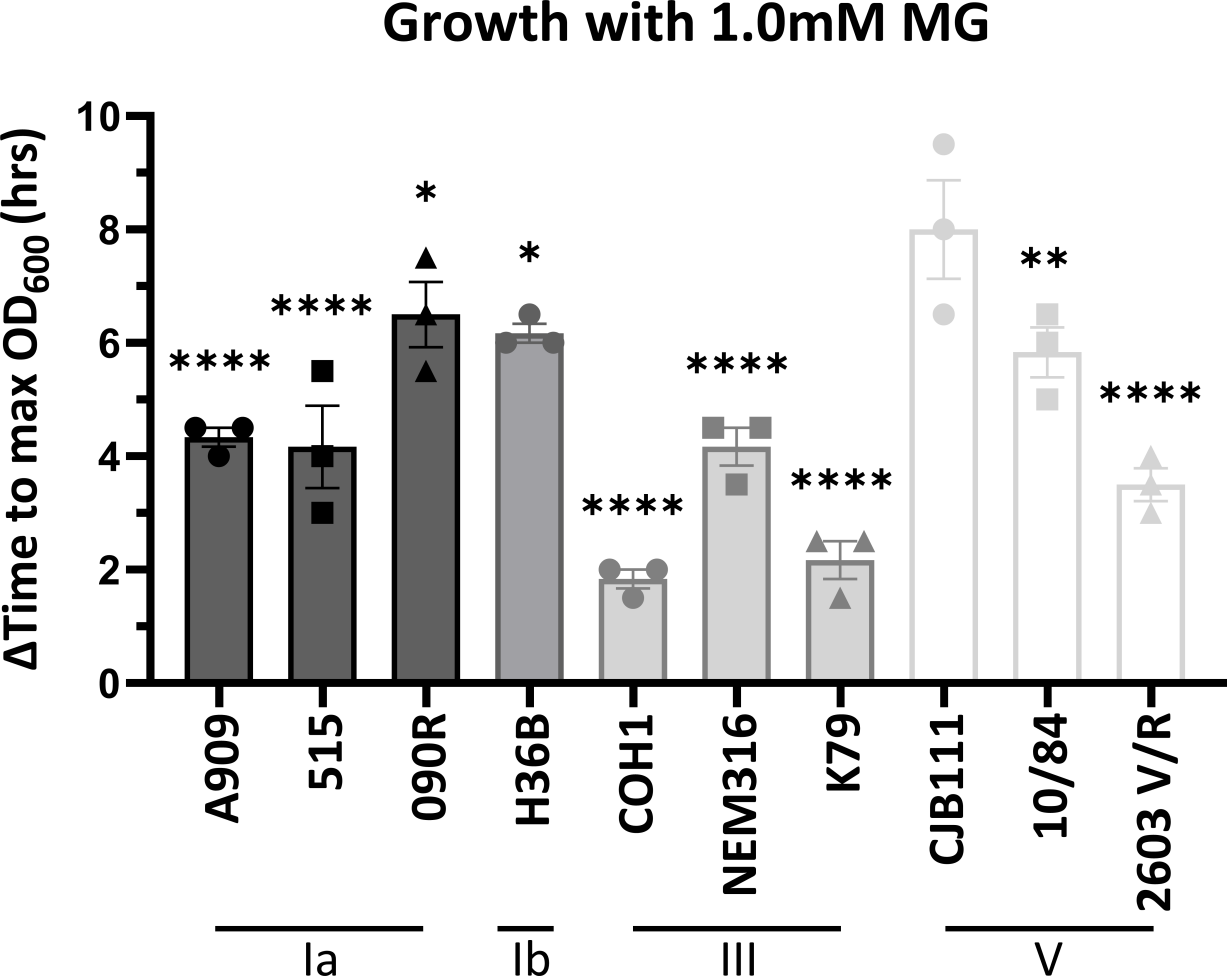
MG sensitivity differs across GBS isolates. Change in time to max OD_600_ for growth curves with 1.0 mM MG compared to no MG in mCDM for representative serotype Ia, Ib, III, and V GBS strains. Significance was determined by comparing all strains to the most sensitive strain, CJB111, by one-way analysis of variance (ANOVA) with uncorrected Fisher’s least significant difference (LSD) test with *P* < 0.05. *, <0.05; **, 0.01; ****, <0.0001.

GBS contains glyoxalase A and B homologs, also known as lactoylglutathione lyase (ID870_02260) and hydroxyacylglutathione hydrolase (ID870_06660), respectively. These are hypothesized to be involved in MG detoxification and therefore, tolerance. To begin to characterize this pathway in GBS, we grew several clinical GBS isolates, representing various capsule serotypes, in the presence of 1.0 mM MG in a modified chemically defined media (mCDM) (64) (Fig. 2). These concentrations of MG resulted in an observed increase in lag phase without a decrease in CFU, suggesting MG has bacteriostatic properties (Fig. S1A). CJB111 exhibited the highest sensitivity to 1.0 mM MG with the largest increase in lag phase quantified by the change in time to max OD_600_ at 8.00 h. All of the strains tested had significantly decreased time to max OD_600_ when compared to CJB111, and COH1 exhibited the lowest sensitivity with the smallest time to max OD_600_ at 1.83 h. Overall, different GBS isolates had varying degrees of resistance to MG, but resistance was not correlated with serotype or growth rate differences (Fig. S1B). To explore the strain differences in MG tolerance further, we selected three representative serotype strains commonly used by our group and others working on GBS pathogenesis with low to high resistance: CJB111 (V), A909 (Ia), and COH1 (III). First, we compared GloA amino acid sequences between these three strains and previously characterized GloA from *Streptococcus pyogenes* and *Escherichia coli* (Fig. S2A) (29, 65–67). We chose these strains since the GAS glyoxalase pathway has been investigated previously and it is within the same genus and the *E. coli* GloA has a solved crystal structure which is important for protein modeling. The CJB111 GloA is 63.5% identical to GAS GloA and 40.5% identical to *E. coli* GloA. Interestingly, the GloA from CJB111 and A909 are 100% identical while the COH1 GloA is only 99% identical due to a single amino acid change of an alanine to a serine (A45S) in a non-conserved region. To determine how common this variant was in GBS, we generated a phylogenetic tree using BlastP and FigTree to compare 57 GBS genomes and found that 12 out of 57 GloA proteins (21%) have the A45S change with another five having a different A45 variant (Fig. S2B). Proteins with the A45S variant also clustered together in the tree suggesting a common ancestral strain, however, only COH1 from the strains tested in Fig. 2 had this variant. To assess tertiary structure, a predicted protein model for GBS GloA was generated using AlphaFold2 and had extremely high confidence for most residues (Fig. S2C and D). The predicted structure was compared to the solved *E. coli* GloA structure (PDB 19FZ) and found to have highly similar topology and conserved metal binding residues (Fig. S2C). GloA was also modeled in its active form as a dimer to show predicted active sites (Fig. S2E Left). The A45S variant from COH1 GloA was included in the dimer and modeled to be next to the predicted active site (Fig. S2E Right). Lastly, using our selected representative strains, we investigated baseline transcription regulation of the glyoxalase pathway by comparing mid-log transcript levels for *gloA* and *gloB* using real-time quantitative polymerase chain reaction (RT-qPCR) and found that COH1 has a higher abundance of both *gloA* and *gloB* transcripts compared to CJB111 and A909 (Fig. S2F). Altogether, the predicted GloA protein in GBS contains conserved residues important for enzyme activity and metal binding but it is not clear if the common A45S amino acid change correlates with enzyme activity. In addition, transcript expression for *gloA* and *gloB* are increased in COH1 which could explain higher MG resistance.

### Glyoxalase A contributes to methylglyoxal detoxification in GBS

**Fig 3:**
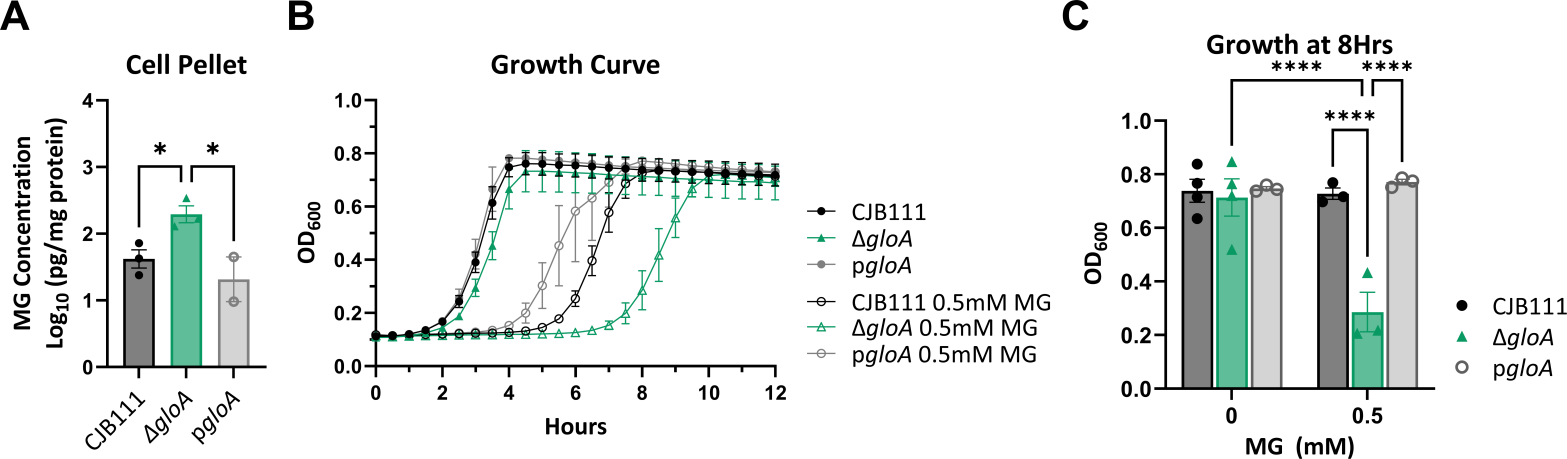
The contribution of glyoxalase A to GBS methylglyoxal detoxification. (**A**) ELISA MG quantification of cell pellets for WT CJB111, Δ*gloA*, and p*gloA* strains after growth in mCDM for 4 h. (**B**) Growth curve measured by OD_600_ for WT CJB111, Δ*gloA*, and p*gloA* strains grown with or without 0.5 mM MG in mCDM. (**C**) Comparison of growth shown in (**B**) at 8 h between strains. Significance determined by (**A**) one-way ANOVA or (**C**) two-way ANOVA with Fisher’s LSD multiple comparisons test with *P* < 0.05. *, <0.05; ****, <0.0001.

To confirm our Tn-sequencing results we chose to study the first enzyme in the glyoxalase pathway, GloA. Using allelic exchange mutagenesis, we constructed a mutant in *gloA* (∆*gloA*) and a complemented strain (p*gloA*) in CJB111 as described in Materials and Methods. MG detoxification was then tested using these strains by MG quantification and growth curve analysis. First, to measure if MG accumulates in the ∆*gloA* strain, we measured MG concentrations using an enzyme-linked immunosorbent assay (ELISA) on lysed cell pellet samples for CJB111 WT, ∆*gloA*, and p*gloA* strains. The concentration of MG was normalized to the total protein concentration of each sample and found to be significantly increased in the ∆*gloA* mutant compared to the CJB111 WT and complemented strains (Fig. 3A). To determine if this accumulated MG in the ∆*gloA* mutant may be toxic/impact GBS growth, all strains were inoculated into mCDM with or without the addition of 0.5 mM MG. Indeed, a greater growth delay was observed for ∆*gloA* with a change in time to max OD_600_ at 4.92 h compared to 3.58 h for WT or 2.83 h for p*gloA*, which confirms *gloA* is involved in MG detoxification (Fig. 3B). Furthermore, the OD_600_ at 8 h was compared between strains and confirmed a significant decrease in ∆*gloA* growth compared to WT or the complemented strain following the addition of MG (Fig. 3C). To determine if the growth delay we observed with MG was due to the emerging resistant subpopulation, we grew CJB111 WT to mid-logarithmic phase with 0.5 mM MG and then inoculated fresh media with or without increasing MG (Fig. S3A). From this experiment, we still observed a growth delay with fresh MG indicating it is not a more resistant subpopulation emerging. As MG is primarily produced from glycolysis in cells (bacteria and host), we further investigated the impact of GloA on GBS growth in mCDM with increasing glucose concentrations. However, we did not observe a growth defect for the ∆*gloA* mutant compared to WT or complemented strain at low (biologically relevant blood glucose concentration), medium (ideal concentration for GBS growth), or high glucose concentrations tested (Fig. S3B). Additionally, upon assessment of general virulence characteristics, we also did not observe a difference in susceptibility to hydrogen peroxide or hemolytic activity between CJB111, ∆*gloA*, and p*gloA* (Fig. S3C and D). Taken together MG quantification and growth analysis suggest that GloA contributes to MG detoxification in GBS. Our results also suggest that, under the conditions tested, GBS may not produce enough MG from glucose metabolism to negatively impact its growth.

### Glyoxalase A is necessary for GBS survival *in vivo*

**Fig 4:**
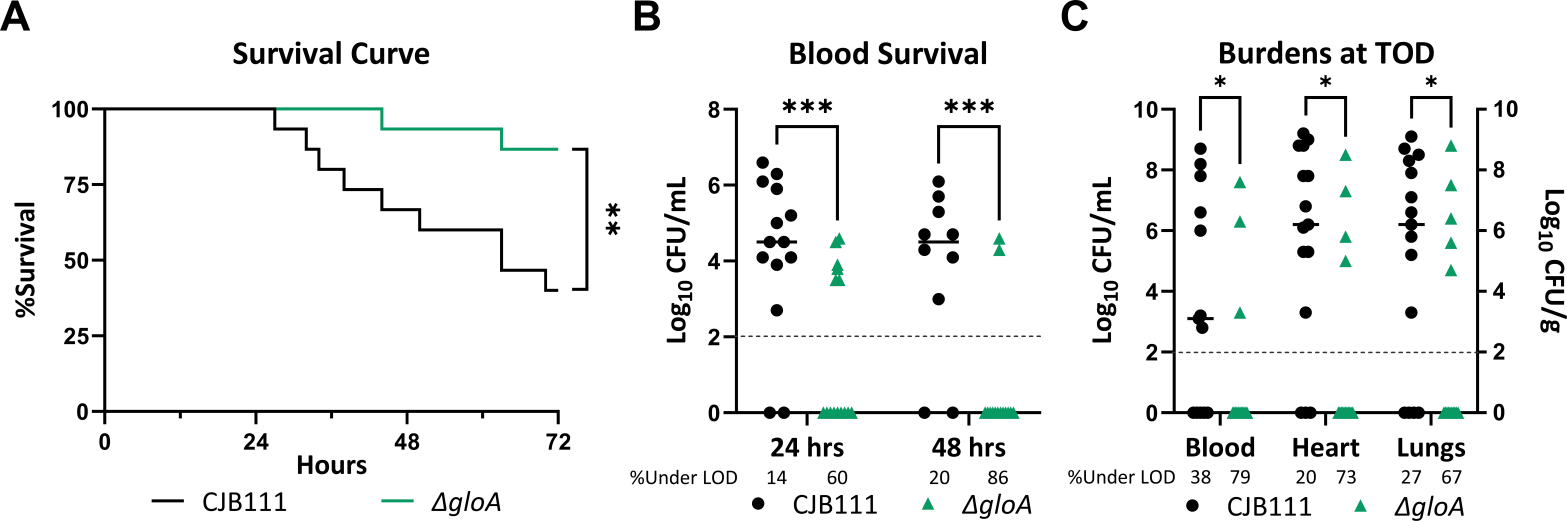
Methylglyoxal detoxification is necessary for GBS infection. (**A**) Survival curve of mice tail-vein injected with 10^7^ CFU WT CJB111 or Δ*gloA*. (**B**) Recovered CFU counts from the blood of infected mice at 24 and 48 h post-infection. (**C**) Recovered CFU counts from the blood, heart, and lungs of infected mice at the time of death. In (B) and (C), the limit of detection is indicated with a dotted line and the percentage of mice with CFU under the limit is shown beneath the bar graphs. Significance determined by (**A**) Log-rank (Mantel-Cox) test, (**B**) mixed-effects analysis with uncorrected Fisher’s LSD test, or (**C**) multiple Mann-Whitney *U* tests with *P* < 0.05. *, <0.05; **, <0.01; ***, <0.001. All mice were included in statistical analyses.

To further confirm the Tn-sequencing results and determine if *gloA* is important during infection, we repeated our bacteremia model of infection by intravenously injecting mice with 1.5–2 × 10^7^ CFU CJB111 WT or the ∆*gloA* mutant and monitoring the infection for up to 72 h post-infection. Mice infected with the ∆*gloA* mutant exhibited significantly decreased mortality compared to those infected with WT, with greater than 75% surviving to the experiment endpoint (Fig. 4A). In order to monitor CFU burden over time, blood samples were taken from surviving mice at 24 and 48 h post-infection and the time of death (TOD) (Fig. 4B). Mice infected with ∆*gloA* had significantly decreased blood burdens as soon as 24 h post-infection and in tissue burdens at the time of death, indicating the mutant strain is not able to survive as well in the bloodstream and disseminate to other organs compared to WT CJB111 (Fig. 4B and C). It is important to note that all of the strains had similar blood burdens at 6 h post-infection (Fig. S4A) and that the ∆*gloA*-infected mice that succumbed to the infection before 72 h had the highest CFU counts in the blood at 24 h (Fig. 4B). Additionally, we infected mice with the complemented strain and confirmed that it was able to survive in the blood longer than ∆*gloA* (Fig. S4).

### Importance of GBS glyoxalase A to neutrophil survival

**Fig 5:**
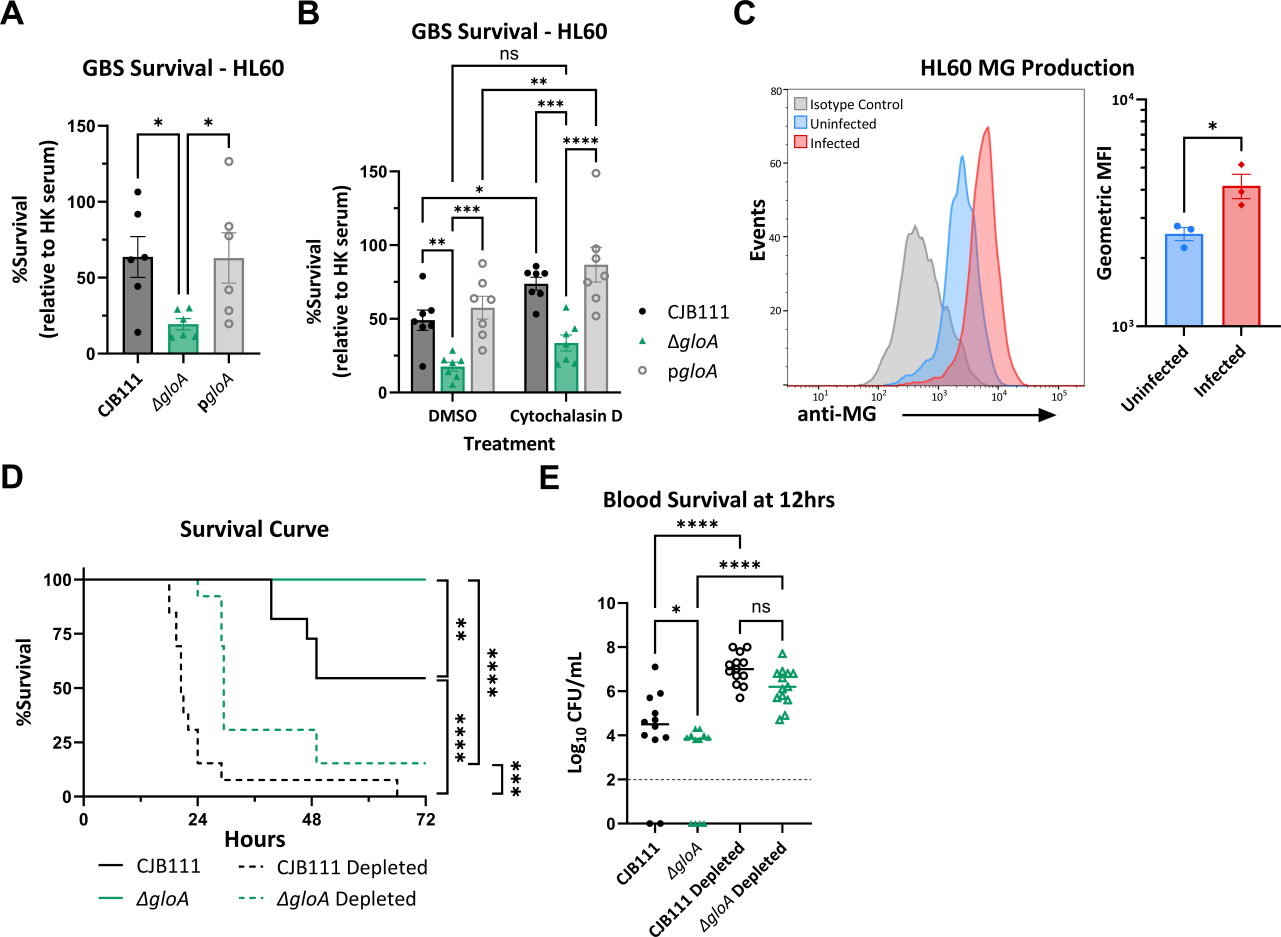
Methylglyoxal detoxification is necessary for GBS survival against neutrophils. Survival of WT CJB111, Δ*gloA*, and p*gloA* strains after 5 h infection of HL60-neutrophils (**A**) without treatment or (**B**) with DMSO control or Cytochalasin D treatment. (**C**) Flow cytometry quantification of intracellular MG-modified proteins in HL60-neutrophils with or without WT CJB111 infection. Left: representative histogram displaying MG signal that includes isotype control, uninfected, and infected cells. Right: geometric MFI quantification for MG in uninfected and infected samples. (**D**) Survival curve of normal or neutrophil-depleted mice that were tail-vein injected with 10^7^ CFU WT CJB111 Δ*gloA*. (**E**) Recovered CFU counts from the blood of infected mice 12 h post-infection. The limit of detection is indicated with a dotted line. Significance determined by (A and E) one-way ANOVA with Fisher’s LSD multiple comparisons test. (**B**) Two-way ANOVA with Fishers LSD multiple comparisons test. (**C**) Unpaired Student *t*-test, (**D**) Log-rank (Mantel-Cox) test with *P* < 0.05. *, <0.05; **, <0.01; ***, <0.001; ****, <0.0001.

Pro-inflammatory M1-type macrophages primarily use glycolysis to generate energy (68) and have been shown to produce aldehydes, such as MG, in response to infection (66, 69–71). However, neutrophils are the primary immune cell GBS encounters during acute infection (55), which also utilize glycolysis as their primary energy source (66, 68–72). Therefore, to determine if GBS *gloA* contributes to neutrophil survival we performed *in vitro* neutrophil killing assays using differentiated HL60-neutrophils with the CJB111 WT, ∆*gloA*, and p*gloA* strains. At 5 h post-infection, the ∆*gloA* mutant strain exhibited significantly decreased survival compared to WT or the complemented strains (Fig. 5A). This phenotype was independent of serum killing and cytotoxicity since serum by itself did not impact GBS survival over time and there were low levels and no differences in HL60 cytotoxicity between the strains at 5 h (Fig. S5A and B). We also confirmed that ∆*gloA* has decreased survival in the presence of primary murine bone marrow neutrophils (BMNs) (Fig. S5C). Next, we used cytochalasin D treatment to block phagocytosis of HL60-neutrophils which resulted in a significant increase in survival for CJB111 WT and p*gloA* strains (Fig. 5B). The ∆*gloA* mutant also had a minor increase in survival with cytochalasin D treatment but it was not significant when compared to the DMSO control and there was still a significant decrease in survival when compared to WT and complement indicating the mutant is susceptible to multiple mechanisms of neutrophil killing. To evaluate if this increased killing might be due to a general increase in neutrophil production of MG in response to GBS infection, we measured the accumulation of intracellular MG-modified proteins in HL60-neutrophils using flow cytometry. Consistent with the literature (30) we observed that all cells contained detectable MG-modified proteins. Interestingly, however, upon infection, we observed a significant increase in anti-MG geometric mean fluorescent intensity (MFI) compared to uninfected controls (Fig. 5C), indicating that infection increases intracellular MG within HL60s. This increase was also only observed in high glucose conditions (Fig. S5D), which is similar to what has been described for *S. pyogenes* where they reported that GloA contributed to survival against neutrophils with elevated glucose (65). To examine the contribution of neutrophils controlling GBS infection *in vivo,* we depleted neutrophils in mice prior to intravenous infection. Mice were injected intraperitoneally with anti-Ly6G or an isotype control 24 h (73) before intravenous infection with 1 × 10^7^ CFU CJB111 or ∆*gloA*. Upon assessing morbidity and mortality of these groups over 72 h post-infection, we observed that both CJB111 and ∆*gloA*-infected neutrophil-depleted mice exhibited a significant increase in mortality when compared to their non-depleted cohorts (Fig. 5D), although percent survival of neutrophil-depleted mice infected with ∆*gloA* remained higher than neutrophil-depleted mice infected with CJB111. At 12 h post-infection, we observed that neutrophil depletion abrogated the attenuated phenotype of the ∆*gloA* mutant in the blood, as the CJB111 and ∆*gloA*-infected, neutrophil-depleted mice did not differ in blood burdens (Fig. 5E). Furthermore, CJB111 and ∆*gloA* CFU burdens were significantly increased in the neutrophil-depleted mice compared to the non-depleted mice. Taken together these results show that the attenuation of the ∆*gloA* mutant can be partially rescued with neutrophil depletion and suggest that MG produced by other cell types may aid in the defense against GBS bloodstream infections.

## DISCUSSION

GBS must be able to survive multiple host niches to cause invasive infections in neonates. Some of these environments include the vaginal tract, amniotic fluid, and blood (1, 74). Tn-sequencing is a powerful and common method for investigating bacterial genes necessary for survival and fitness in these different environments. Previously, an *ex vivo* Tn-sequencing was performed in human blood using a TnSeq library in the GBS A909 serotype Ia background (75). Their results found similar underrepresented genes compared to our *in vivo* data set such as genes involved in capsule biosynthesis, metal homeostasis, and arginine metabolism. Interestingly, they identified *relA* to be underrepresented, which encodes a GTP pyrophosphokinase and is a central regulator of the stringent response in GBS. They found that *relA* not only controls stringent response activation and the arginine deiminase pathway but also impacts βH/C production. While we did not observe *relA* in our data set, other putative stress response proteins like *ytgB* and *asp1* were significantly underrepresented. Most notably, *asp1* is annotated as an Asp^23^/Gls^24^ family envelope stress response protein and was found to be upregulated when GBS was incubated in human blood (13, 76) and downregulated after exposure to high glucose (77). We also observed that the two-component system (TCS) *dltRS* and a *dlt* gene were underrepresented, which are involved in modulating surface charge and contribute to cationic antimicrobial peptide resistance and decrease phagocytic killing (35, 54). Previous studies have shown that a *dltA* mutant exhibited decreased virulence in a murine model with significantly lower burdens in tissue and blood compared to WT GBS (35). Another TCS, *bceRS/nsrRK,* exhibited the highest negative fold change of the underrepresented TCS and has been shown to contribute to bacitracin (antibiotic), nisin (lantibiotic), cathelicidin/LL-37 (human antimicrobial peptide), and oxidative stress resistance (56, 57). Its role in GBS pathogenesis was demonstrated as a *bceR* mutant yielded decreased virulence during murine infection and decreased biofilm formation (56). Another top gene hit identified within the present study to be important for GBS blood survival is the C5a peptidase *scpB. scpB* is already known to be involved in complement evasion and fibronectin binding and is associated with neonatal isolates (36, 78). Overall, identifying these known virulence factors in our study demonstrates the validity of our *in vivo* Tn-sequencing screen to identify novel factors important for blood survival in mice and supports previous research in the streptococcal field. It also provides another resource for developing new hypotheses and research projects. For example, MG detoxification has not been previously characterized in prior GBS studies.

MG is a highly reactive electrophilic species (RES) and a byproduct of normal cell metabolism which can be spontaneously or enzymatically produced by all cells (28, 30) with up to 90% of cell MG estimated to come from glycolysis alone (79). Notably, MG is also a precursor to advanced glycation end products (AGEs) and is associated with many other human diseases like diabetes, cancer, and neurological disorders like Alzheimer’s disease (30, 80). The most well-known and ubiquitous pathway for MG detoxification is the glyoxalase pathway which consists of glyoxalase A (*gloA*) and glyoxalase B (*gloB*) enzymes. Recently, the glyoxalase pathway, especially *gloA*, in *Listeria monocytogenes* was found to contribute to intracellular survival in macrophages and during murine infection (66). In addition, the glyoxalase pathway in *S. pyogenes* was shown to be important for survival against neutrophils in a glucose and myeloperoxidase-dependent manner (65). The GBS *gloA* and *gloB* homologs were underrepresented in our Tn-sequencing data set, therefore, we hypothesized that GBS may encounter host-derived MG during bacteremia as a response to infection (65, 69, 81, 82). The first step in the glyoxalase pathway is mediated by GloA and, in this study, we have characterized its contribution to GBS MG tolerance *in vitro* and infection *in vivo*. We observed that a Δ*gloA* mutant exhibited decreased survival in the presence of HL60-neutrophils as well as primary murine neutrophils and that its virulence was largely restored in neutrophil-depleted mice. We also measured an increase in MG-modified proteins in HL60-neutrophils upon GBS infection which is likely from increased production of MG by the cells themselves. These results indicate that MG-mediated killing may constitute another important defense mechanism used by immune cells to kill invading bacteria.

We also observed that tolerance to MG varies across GBS isolates and is not definitively correlated to serotype, GloA amino acid sequence, or glyoxalase gene regulation. Of note, two of the serotype III strains, the most commonly isolated serotype from neonatal invasive infections (83), had the first and second highest overall tolerance to MG, but additional strains would need to be tested to determine if serotype III has higher average MG tolerance. Interestingly, COH1 (serotype III), which had the highest resistance to MG, was the only strain tested that had the A45S variant in GloA and higher baseline transcript levels of *gloA* and *gloB* compared to CJB111 and A909 strains. The A45S variant is also only 10 amino acids away from the K55 residue which is predicted to be involved in metal binding and could impact folding or metal coordination (67, 84, 85). However, other GBS strains tested also had high resistance to MG, like K79, which does not have the A45S variant. Therefore, it is unlikely that this amino acid change is the sole determinant of enzyme activity, but further investigation is needed to determine if GloA protein variants and regulation impact GBS MG tolerance. Relatedly, *Streptococcus mutans* was shown to be more tolerant to MG than most other commensal oral Streptococcal species and was also shown to outcompete *Streptococcus sanguinis* when MG was present in a competition experiment (86). From this previous study and our work shown here, we hypothesize that differences in GBS MG tolerance could be influenced by the environments they were found in, like the presence of other bacterial species or host differences.

Components of metal transport systems were also significantly underrepresented in the Tn-sequencing data set with top hits including the zinc import system *adcAAIIBC* and *lmb,* the manganese import system *mtsABC,* and the putative nickel import system *nikABCDE* (Table 1). These results suggest GBS requires trace metals to survive in the blood. Previously it has already been shown that zinc and manganese transporters are important for maintaining GBS metal homeostasis and contribute to vaginal colonization and female reproductive tract ascension as well as blood survival (13, 32, 76, 87). In addition, both zinc and manganese import systems are important in combating nutritional immunity, or the sequestration of nutrients by the host, mediated by a neutrophil-produced metal-binding protein called calprotectin (33, 46). The nickel transporter, however, has not been as well characterized. In our previous study, we attempted to measure nickel concentrations in a *nikA* mutant, but it was under the limit of detection in our samples. However, we did observe lower levels of copper indicating the system could be transporting more than one metal (47). There are only nine known enzymes present in archaea, bacteria, plants, and primitive eukaryotes that are nickel-dependent, with urease being the most notorious, however, GBS does not encode a urease enzyme (32, 47, 88, 89). Interestingly, another of the nine known nickel-dependent enzymes is GloA which was found to use nickel (Ni^2+^) as a cofactor in *E. coli* (34, 67, 85). Therefore, the importance of the nickel transporter in the blood could be due to increased GloA activity but the requirement for nickel in GBS remains to be elucidated.

MG is formed primarily from glycolysis but it can be produced, albeit to a lesser extent, from lipid, ketone, and protein metabolism (30, 34). MG is toxic to cells due to its electrophilic properties allowing it to react with different molecules, like DNA and protein, and effectively arrest growth (81). For example, MG has been shown to increase mutation rates in *L. monocytogenes* by binding DNA (66) and inhibits protein synthesis and modification by binding to guanine and arginine residues (90–93). It is important to note that in some bacteria, like *E. coli* and *L. monocytogenes,* MG can be formed directly from dihydroxyacetone phosphate during glycolysis by methylglyoxal synthase (*mgsA*); however, GBS, like other streptococci, do not have an MG synthase gene (65, 86). The lack of a synthase gene further supports our hypothesis that GBS encounters host-derived MG toxicity during infection. Since approximately 99% of cellular MG is thought to be already bound to molecules like DNA and protein it is difficult to quantify accurately; however, intracellular MG concentrations are consistently estimated below 10 µM and are known to be dependent on glutathione concentrations (30, 94, 95). In the serum of diabetic individuals, the concentration of MG and MG-derived AGEs is increased compared to healthy individuals, most likely due to increased glucose concentrations (95). MG is historically tied to diabetes because it is known to exacerbate diabetic complications like microvascular and kidney dysfunction and contribute to the progression of the disease (95). Our lab has shown that GBS is a common colonizer of infected diabetic wounds (96) and we show here the production of MG-modified proteins by HL60-neutrophils is dependent on glucose and GBS infection (Fig. 5C; Fig. S5D). Therefore, research into the role of the GBS glyoxalase pathway in the context of diabetic wound infection is a current area of study.

Lastly, the conversion of MG to D-lactate by the glyoxalase pathway was first described over 100 years ago and is the most ubiquitous and conserved process for MG detoxification across all kingdoms of life (28). MG detoxification has not been thoroughly studied in streptococci in the context of disease and has never been characterized in GBS. Thus far, studies focusing on *S. pyogenes*, *S. mutans*, and *S. sanguinis* have shown *gloA* to be the primary modulator of MG tolerance *in vitro* with *gloB* mutants having little effect (65, 86). This is in support of what was observed with *L. monocytogenes,* but not *Salmonella* where it was found that *gloB* was more important for *Salmonella* resistance to oxidative stress and killing by macrophages (66, 97). Here, we found *gloA* to be dispensable to GBS tolerance of H_2_O_2_ (Fig. S3C) but the contribution of *gloB* remains to be examined. Additional enzymes known to break down MG into acetol or lactaldehyde intermediates include aldose, aldehyde, and MG reductases. A putative aldo/keto reductase (*yvgN*) was significantly underrepresented in our data set (Table 1) but its contribution to GBS virulence requires further investigation (34).

In this study, we demonstrate, for the first time, the essential role of the glyoxalase pathway in GBS MG resistance and overall pathogenicity during bloodstream infection. We investigated the role of GloA *in vitro* and *in vivo* and confirmed it is important for growth in the presence of MG, survival against neutrophils, and during invasive infection. Our study also provides further evidence in support of the aldehyde hypothesis that MG detoxification is an important component for bacterial survival against host defenses; however, the role of the glyoxalase system in GBS survival in macrophages requires further investigation (69). Specifically, we show increased MG production by neutrophils in response to infection. Overall, research aimed at understanding metabolic mechanisms used by bacteria to survive in the blood and RES toxicity will be important for the development of new treatments and therapies for infection and will expand our knowledge about host-pathogen interactions.

## MATERIALS AND METHODS

### Bacterial strains, media, and growth conditions

See Table S2 for strains and primers used in this study. GBS strains were grown statically at 37°C in Todd-Hewitt Broth (THB) unless otherwise stated. Streptococcal chemically defined medium (64) was modified by omitting L-cysteine and adding 22 mM glucose unless otherwise stated. *Escherichia coli* strains for cloning were grown in LB at 30°C or 37°C with rotation at 250 rpm. Kanamycin and erythromycin (Sigma-Aldrich, St. Louis, MO) were supplemented to media at 50 µg/mL and 500 µg/mL, respectively, for *E. coli*. Kanamycin, spectinomycin, and erythromycin (Sigma-Aldrich, St. Louis, MO) were supplemented to media at 500 µg/mL, 100 µg/mL, and 5 µg/mL, respectively, for streptococcal strains.

### Routine molecular biology techniques

All PCR reactions utilized Phusion or Q5 polymerase (Thermo Fisher, Waltham, MA). PCR products and restriction digest products were purified using a QIAquick PCR purification kit (Qiagen, Venlo, NL) per manufacturer protocols. Plasmids were extracted using the QIAprep miniprep kit or plasmid midi kit (Qiagen, Venlo, NL) per manufacturer protocols. Restriction enzyme digests utilized XmaI, EcoR1, and BamH1 (New England Biolabs, Ipswich, MA) for 2 h at 37°C in a thermocycler. Ligations utilized Quick ligase (New England Biolabs, Ipswich, MA) at room temperature for 5 min or Gibson Assembly Master Mix (New England Biolabs, Ipswich, MA) per manufacturer protocols. All plasmid constructs were sequence confirmed by Sanger sequencing (CU Anschutz Molecular Biology Core, Aurora, CO) or whole plasmid sequencing (Quantara Biosciences, Hayward, CA).

The mutant strains were generated as previously described (12, 32). Briefly, for the *gloA* mutant, genomic 5′ and 3′ regions flanking the *gloA* gene were amplified and fused with a spectinomycin cassette by FailSafe PCR (Lucigen, Middleton, WI). Fragments and pHY304 vector were digested with restriction enzymes and ligated using Quick Ligase. The ligation reaction product was transformed into chemically competent *E. coli*. pHY304 plasmids were purified from *E. coli* and electroporated into GBS CJB111 genetic background. Constructs were confirmed by PCR and sequencing. Complement strains were generated by amplifying the *gloA* gene in GBS and linearizing pABG5 by PCR. Products were ligated using Gibson assembly and then transformed into chemically competent *E. coli*. Plasmids were purified from *E. coli* and electroporated into GBS CJB111 Δ*gloA* genetic background. Primers used in the construction of strains are listed in Table S2. The mutant had no growth or hemolysis defects observed (Fig. S3B through D).

### Study approval

Animal experiments were approved by the Institutional Animal Care and Use Committee at the University of Colorado Anschutz Medical Campus protocol #00316 and were performed using accepted veterinary standards. The University of Colorado Anschutz Medical Campus is AAALAC accredited, and its facilities meet and adhere to the standards in the “Guide for the Care and Use of Laboratory Animals.” All mice were purchased from Charles River Laboratories (CD1) and housed in pathogen-free, biosafety level-2 animal facilities.

### *In vivo* transposon screening

Triplicate cultures of the pooled CJB111 pKrmit transposon library (32) were grown overnight at 37°C in THB with kanamycin at 300 µg/mL and back diluted to an OD600 0.4. Libraries were normalized to ~4 × 10^7^ CFU/100 µL and injected via tail-vein into 6–8-week-old CD-1 male mice using the established hematogenous infection model (98–101). Blood was collected by cardiac puncture between ~18 and 28 h post-infection. A 100 µL of input library and blood was plated in duplicated on CHROMagar Strep B with 300 µg/mL kanamycin and incubated overnight at 37°C to collect recovered transposon mutants. Bacterial growth from spread plates was collected and 3–4 mice per library were pooled together, genomic DNA was extracted using a ZymoBiomics DNA miniprep Kit (Zymo Research).

### Transposon library sequencing

Libraries were prepared and sequenced at the University of Minnesota Genomics Center according to https://www.protocols.io/view/transposon-insertion-sequencing-tn-seq-library-pre-rm7vzn6d5vx1/v1. Briefly, genomic DNA was enzymatically fragmented, and adapters added using the NEB Ultra II FS kit (New England Biolabs), and ~50 ng of fragmented adapted gDNA was used as a template for enrichment by PCR (16 cycles) for the transposon insertions using mariner-specific (TCGTCGGCAGCGTCAGATGTGTATAAGAGACAGCCGGGGACTTATCATCCAACC) and Illumina P7 primers. The enriched PCR products were diluted to 1 ng/uL and 10 uL was used as a template for an indexing PCR (nine cycles) using Nextera_R1 (iP5) and Nextera_R2 (iP7) primers. Sequencing was performed using a 150-base paired-end format on an Illumina NextSeq 2000 and Illumina NovaSeq 6000 system to generate ~40–60 million reads per library.

### Tn-sequencing analysis

The R1 reads from both sequencing runs were concatenated and quality was assessed using FastQC (102) (http://www.bioinformatics.babraham.ac.uk/projects/fastqc/). Reads were trimmed using Cutadapt (v 4.2) (103) with the following parameters; sequence length with a minimum of 12 bases, removal of flanking “N” bases, reads trimmed of 3′ “G” bases, and reads were trimmed with the reverse complemented mariner transposon sequence (ACTTATCAGCCAACCTGTTA). TRANSIT (v 3.2.7) (104) was used to align trimmed reads to the CJB111 genome (CP063198) and for analysis of transposon insertion sites. The Transit PreProcessor processed reads using default parameters with the Sassetti protocol, no primer sequence, and mapped to the genome sequence using Burrows-Wheeler Alignment (105). Insertion sites were normalized using the Total Trimmed Reads method in TRANSIT and analyzed using the resampling method to compare the insertion counts recovered in blood vs the input library using default parameters, with the addition of ignoring TA sites within 5% of the 5′ and 3′ end of the gene. Significance determined by *P*_adj_ < 0.05 and log_2_FC < −1 or >1. All sequencing reads have been deposited into NCBI SRA under BioProject ID PRJNA1125445.

### Murine model of bloodstream infection

We infected mice as previously described for CJB111 to cause invasive infection (98–101). Briefly, fifteen 8-week-old CD1 male mice were intravenously challenged with 1.5–2 × 10^7^ CFU GBS CJB111, ∆*gloA,* or p*gloA*. At 6, 12, 24, and/or 48 h post-infection, blood samples were taken by tail prick and plated on THA to quantify GBS CFU burden. Once mice reached a moribund state or 72 h post-infection mice were sacrificed, and blood was harvested by cardiac puncture, and lung and heart tissue were removed and homogenized in sterile phosphate buffered saline (PBS). All samples were plated on THA or CHROMagar to quantify GBS CFU burden. For neutrophil depletion, 11–13 mice per group were given 200 µg *InVivo*MAb anti-mouse Ly6G antibody (Bio X Cell, Lebanon, NH) or 200 µg *InVivo*MAb rat IgG2a isotype control diluted in *InVivo*Pure pH 7.0 dilution buffer by intraperitoneal injection 24 h before infection.

### GloA protein comparisons

GloA amino acid sequences were aligned using ClustalOmega (106) and the alignment figure was created using the ESPript Server (107) (https://espript.ibcp.fr). Protein IDs used: ABA45143.1 (GBS A909), QOW77196.1 (GBS CJB111), WP_001116201.1 (GBS COH1), WP_002985686.1 (GAS 5448), and P0AC81.1 (*E. coli* K-12). GBS GloA phylogenetic tree was generated using NCBI BlastP (108, 109) and visualized using FigTree v1.4.4 (http://tree.bio.ed.ac.uk/software/figtree/). The protein ID QOW77196.1 (GBS CJB111) was used as the query against *S. agalactiae* and only proteins with a percent identity and query cover greater than 50% are shown. The dimeric structure of the *S. agalactiae* GloA was predicted using AlphaFold2 (110) as implemented in ColabFold (111). PyMOL (version 2.5.2, Schrödinger, LLC.) was used to create images of the predicted GloA structure and the *E. coli* glyoxalase I crystal structure RCSB PDB entry 19FZ (67, 112) (RCSB.org).

### *In vitro* growth comparisons

Overnight cultures of Streptococcal strains were diluted 1:100 in mCDM with or without methylglyoxal (MG, Sigma-Aldrich M0252, St. Louis, MO) or hydrogen peroxide 3% wt/wt (VWR, Radnor, PA) at concentrations listed in figure legends in a 96-well plate. For strain/serotype growth comparisons with MG, the overnight cultures were first normalized to OD600 0.6 in PBS before being diluted to 1:100 in mCDM with or without MG. For testing the effects of MG pre-exposure on growth, overnight cultures were diluted 1:100 in mCDM with 0.5 mM MG and allowed to grow until the mid-logarithmic phase (OD600 0.4–0.6). Then the mid-logarithmic cultures were used to inoculate a new 96-well plate with fresh mCDM with or without MG. For all growth curves longer than 8 h the plate was covered with a Breathe-Easy gas permeable sealing membrane (USA Scientific, Ocala, FL) and then incubated at 37°C without shaking in a Tecan Infinite M Plex for up to 24 h with OD600 taken every 30 min. For growth curves with CFU/mL shown, the 96-well plate was incubated at 37°C without shaking and samples were taken every 2 h for dilution plating on THA. All growth curves were performed in biological triplicate.

### ELISAs on culture pellets

Overnight cultures of Streptococcal strains were diluted 1:100 in mCDM and then grown for 4 h at 37°C. A 3 mL of each culture was pelleted, re-suspended in PBS, and then homogenized using 0.1 mm dia. Zirconia beads. Methylglyoxal concentration in culture samples was measured using an ELISA kit (Biomatik EKN53482, Kitchener, ON, CA) per manufacturer instructions. Protein concentration in each culture was quantified by Bradford assay, using bovine serum albumin (BSA) standard, and each group was performed in biological duplicate or triplicate.

### RT-qPCR

Samples were made by centrifuging 1 mL aliquots of cultures grown to mid-log phase in mCDM and then re-suspending in 1 mL fresh mCDM and incubating for 30 min at 37°C. A 1 mL of RNAProtect Bacteria Reagent (Qiagen, Venlo, NL) was then added before centrifuging and washing pellets with ice-cold PBS. Sample RNA was prepped using the NucleoSpin RNA kit (Macherey-Nagel, Dueren, DE) and TURBO DNase treated (Invitrogen by Thermo Fisher, Waltham, MA) per manufacturer instructions. A 250 ng of RNA was made into cDNA for each sample using the qScript cDNA Synthesis Kit (Quantabio, Beverly, MA) per manufacturer instructions. cDNA was then diluted 1:20 in water and RT-qPCR run using PerfeCTa SYBR Green FastMix (Quantabio, Beverly, MA) per manufacturer instructions and *glcK*, *gloA*, and *gloB* qPCR primers (see Table S2). Each sample was run in technical duplicate for each gene. Each sample Cq value for *glcK*, *gloA*, and *gloB* was normalized to the total average CJB111 Cq value for each gene, respectively.

### Hemolysis assay

Overnight cultures of Streptococcal strains were diluted 1:100 in THB and then grown to mid-log phase at 37°C. Cultures were then normalized to OD600 0.4 in PBS. A volume of 400 µL blood, 400 µL PBS, and 200 µL normalized culture was added to each sample microfuge tube. A volume of 400 µL blood and 600 µL sterile water was added to positive control tubes and 400 µL blood and 600 µL PBS was added to negative control tubes. Tubes were made in technical duplicate and incubated at 37°C with rotation. At 24 h, 100 µL aliquots were taken and centrifuged at 5,500 × *g* for 1 min. OD543 of supernatant was measured using a Tecan Infinite M Plex and %lysis was calculated by subtracting negative control from all samples and then dividing samples by positive controls.

### HL60-neutrophil and primary bone marrow neutrophil-killing assays

HL60 cells were cultured in RPMI + 10% FBS, differentiated with 1.25% DMSO (Sigma-Aldrich, St. Louis, MO) for 4 days, and infected as previously described (113). Briefly, GBS strains were grown to mid-log, normalized in PBS, and then opsonized in 10% normal human serum or heat-killed (HK) serum in FBB buffer (0.5% BSA and 2.2 mM CaCl_2_ in HBSS) for 15 min in a 96-well plate. HL60 cells were diluted to the desired concentration in FBB buffer and then either pre-incubated with 20 µM cytochalasin D (Sigma-Aldrich C8273, St. Louis, MO) or DMSO (Sigma-Aldrich, St. Louis, MO) vehicle control for 15 min with rotation or immediately added to each well with opsonized bacteria to reach an MOI of 0.002. The plate was incubated at 37°C with shaking for 5 h. %Survival at 5 h was calculated by dividing CFU recovered from wells with GBS opsonized with normal serum by CFU recovered from wells with GBS opsonized with HK serum. CFU from control wells without HL60 cells were also quantified.

Murine neutrophils were isolated from the bone marrow of 8–12-week-old C57B6/J mice using an Anti-Ly-6G Microbeads kit (Miltenyi Biotec 130-120-337, Germany) per the manufacturer’s instructions. Neutrophil isolations yielded ≥95% purity and were used within 1 h of isolation. Murine BMNs were re-suspended in RPMI + 5% FBS and then adhered to TC-treated 96-well plates for 30 min prior to infection. Neutrophils were infected at an MOI of 0.01 and then synchronized by centrifugation (200 × *g* for 5 min). Neutrophils were incubated for 4 h in a cell culture incubator at 37°C with a constant rate of 5% CO_2_. At time points, supernatants were removed and neutrophils were washed with DPBS. Neutrophils were then incubated with 2% Saponin in PBS for 12 min and then dilution plated on THA to enumerate GBS CFU/well. To calculate %associated, the recovered CFU/well for all biological replicates of WT CJB111 at 4 h were averaged and then each biological replicate for each strain was normalized to the WT average.

### Cytotoxicity assay

Cytotoxicity of HL60-neutrophils after infection with GBS strains was determined by LDH release using the CyQUANT LDH Cytotoxicity Assay (Invitrogen by Thermo Fisher C20301, Waltham, MA). Briefly, HL60-neutrophils were infected as described in the paragraph above, and then at 5 h, 100 µL of supernatant from each well was removed and spun down at 500 × *g* for 1 min to remove cells and debris. Then the kit was performed as per the manufacturer’s instructions.

### Flow cytometry detection of methylglyoxal-modified proteins in HL60 cells

To determine the impact of infection on methylglyoxal levels in HL60-neutrophils, differentiated Hl60s were first re-suspended in fresh RPMI + 10% FBS with 0 or 20 mM glucose and allowed to equilibrate for 2 h. PBS or WT GBS at MOI 20 was added to 1 mL aliquots of 10^6^ differentiated HL60 cells and incubated at 37°C with rotation for 2.5 h and then harvested by centrifugation (500 × *g*). Cells were then stained using eBioscience Fixable Viability Dye eFluor 506 (Catalog # 65-0866-18) in PBS for 30 min at room temperature followed by anti-human Cd11b antibody conjugated to FITC (1:20 dilution; BD Biosciences 562793) in MACS buffer (1.25 g BSA, 0.185 g EDTA, 250 mL PBS) for 30 min at room temperature. Cells were fixed and permeabilized using the FoxP3 fixation/permeabilization kit (Thermo Fisher Scientific, Catalog # 00-5523-00) according to manufacturer’s instructions before staining for intracellular methylglyoxal using an anti-MG antibody conjugated to PE (clone 9E7; Cat # MA5-45812; recognizes methylglyoxal-modified proteins) or an IgG2a isotype control (Cat # MG2A04) at final concentrations of 0.67 ug/mL (30 min in permeabilization buffer at room temperature). Stained cells were run on a BD LSRFortessa (BD Biosciences) using the BD FacsDiva software (v9) and analyzed by BD FlowJo software (v10.8). Gating strategy was determined by fluorescence minus one control. Flow cytometric histograms display anti-MG staining of 2000 CD11b+ events per sample.

### Statistical analysis

Statistical analysis was performed using Prism version 10.1 for Windows (GraphPad Software, San Diego, CA, USA) as described in figure legends.

## SUPPLEMENTAL MATERIAL

Fig. S1 iai.00540-24-s0001.tif Growth curves in modified CDM with different GBS strains.

Fig. S2 iai.00540-24-s0002.tif GloA protein characterization in GBS.

Fig. S3 iai.00540-24-s0003.tif Impact of MG and Glyoxalase A on GBS growth and virulence.

Fig. S4 iai.00540-24-s0004.tif *In vivo* infection with complemented strain and early blood timepoint.

Fig. S5 iai.00540-24-s0005.tif Impact of serum, primary cells, and glucose on neutrophil phenotypes.

Supplemental material iai.00540-24-s0006.docx Legends for supplemental figures and tables.

Table S1 iai.00540-24-s0007.xlsx Complete *in vivo* blood Tn-sequencing dataset.

Table S2 iai.00540-24-s0008.xlsx Primers and strains.

ASM does not own the copyrights to Supplemental Material that may be linked to, or accessed through, an article. The authors have granted ASM a non-exclusive, world-wide license to publish the Supplemental Material files. Please contact the corresponding author directly for reuse.

## References

[1] Doran KS, Nizet V. 2004. Molecular pathogenesis of neonatal group B streptococcal infection: no longer in its infancy. Mol Microbiol 54:23–31. doi:10.1111/j.1365-2958.2004.04266.x 15458402

[2] Francois Watkins LK, McGee L, Schrag SJ, Beall B, Jain JH, Pondo T, Farley MM, Harrison LH, Zansky SM, Baumbach J, Lynfield R, Snippes Vagnone P, Miller LA, Schaffner W, Thomas AR, Watt JP, Petit S, Langley GE. 2019. Epidemiology of invasive group B streptococcal infections among nonpregnant adults in the United States, 2008-2016. JAMA Intern Med 179:479. doi:10.1001/jamainternmed.2018.7269 30776079 PMC6450309

[3] El-Gendy A-EA, Hassan SET, Gertz B, Bernard B, Ahmed MM, Elzohry HA, Abd El Tawab G, El-Sayed ME-T, Kamel SY, Zakaria H, Mahros AM, Ahmed MH, Tahoon MA, Sakr MA, Gadallah AA-A, Ahmed FE, Elgazzar MF. 2021. Serotyping and antibiotic susceptibility of invasive Streptococcus agalactiae in Egyptian patients with or without diabetes mellitus. Am J Trop Med Hyg 105:1684–1689. doi:10.4269/ajtmh.21-0300 34607308 PMC8641333

[4] Edwards MS, Rench MA, Haffar AA, Murphy MA, Desmond MM, Baker CJ. 1985. Long-term sequelae of group B streptococcal meningitis in infants. J Pediatr 106:717–722. doi:10.1016/s0022-3476(85)80342-5 3889248

[5] Libster R, Edwards KM, Levent F, Edwards MS, Rench MA, Castagnini LA, Cooper T, Sparks RC, Baker CJ, Shah PE. 2012. Long-term outcomes of group B streptococcal meningitis. Pediatrics 130:e8–e15. doi:10.1542/peds.2011-3453 22689869

[6] Hall J, Adams NH, Bartlett L, Seale AC, Lamagni T, Bianchi-Jassir F, Lawn JE, Baker CJ, Cutland C, Heath PT, Ip M, Le Doare K, Madhi SA, Rubens CE, Saha SK, Schrag S, Sobanjo-Ter Meulen A, Vekemans J, Gravett MG. 2017. Maternal disease with group B streptococcus and serotype distribution worldwide: systematic review and meta-analyses. Clin Infect Dis 65:S112–S124. doi:10.1093/cid/cix660 29117328 PMC5850000

[7] Kurian NK, Modi D. 2022. Mechanisms of group B Streptococcus-mediated preterm birth: lessons learnt from animal models. Reprod Fertil 3:R109–R120. doi:10.1530/RAF-21-0105 35794927 PMC9254271

[8] Berardi A, Trevisani V, Di Caprio A, Bua J, China M, Perrone B, Pagano R, Lucaccioni L, Fanaro S, Iughetti L, Lugli L, Creti R. 2021. Understanding factors in group B Streptococcus late-onset disease. Infect Drug Resist 14:3207–3218. doi:10.2147/IDR.S291511 34429620 PMC8380284

[9] Sabroske EM, Iglesias MAS, Rench M, Moore T, Harvey H, Edwards M, Baker CJ, Flores AR. 2023. Evolving antibiotic resistance in group B streptococci causing invasive infant disease: 1970-2021. Pediatr Res 93:2067–2071. doi:10.1038/s41390-022-02375-3 36352262 PMC10166762

[10] Vornhagen J, Adams Waldorf KM, Rajagopal L. 2017. Perinatal group B streptococcal infections: virulence factors, immunity, and prevention strategies. Trends Microbiol 25:919–931. doi:10.1016/j.tim.2017.05.013 28633864 PMC5650539

[11] Manzer HS, Nguyen DT, Park JY, Park N, Seo KS, Thornton JA, Nobbs AH, Doran KS. 2022. The group B streptococcal adhesin BspC interacts with host cytokeratin 19 to promote colonization of the female reproductive tract. MBio 13:e0178122. doi:10.1128/mbio.01781-22 36069447 PMC9600255

[12] Spencer BL, Tak U, Mendonça JC, Nagao PE, Niederweis M, Doran KS. 2021. A type VII secretion system in group B Streptococcus mediates cytotoxicity and virulence. PLoS Pathog 17:e1010121. doi:10.1371/journal.ppat.1010121 34871327 PMC8675928

[13] Mereghetti L, Sitkiewicz I, Green NM, Musser JM. 2008. Extensive adaptive changes occur in the transcriptome of Streptococcus agalactiae (group B streptococcus) in response to incubation with human blood. PLoS One 3:e3143. doi:10.1371/journal.pone.0003143 18769548 PMC2519835

[14] Akbari MS, Doran KS, Burcham LR. 2022. Metal homeostasis in pathogenic streptococci. Microorganisms 10:1501. doi:10.3390/microorganisms10081501 35893559 PMC9331361

[15] Liu Y, Liu J. 2022. Group B streptococcus: virulence factors and pathogenic mechanism. Microorganisms 10:2483. doi:10.3390/microorganisms10122483 36557736 PMC9784991

[16] Spellerberg B, Pohl B, Haase G, Martin S, Weber-Heynemann J, Lütticken R. 1999. Identification of genetic determinants for the hemolytic activity of Streptococcus agalactiae by ISS1 transposition. J Bacteriol 181:3212–3219. doi:10.1128/JB.181.10.3212-3219.1999 10322024 PMC93778

[17] Doran KS, Liu GY, Nizet V. 2003. Group B streptococcal beta-hemolysin/cytolysin activates neutrophil signaling pathways in brain endothelium and contributes to development of meningitis. J Clin Invest 112:736–744. doi:10.1172/JCI17335 12952922 PMC182187

[18] Ring A, Braun JS, Pohl J, Nizet V, Stremmel W, Shenep JL. 2002. Group B streptococcal beta-hemolysin induces mortality and liver injury in experimental sepsis. J Infect Dis 185:1745–1753. doi:10.1086/340818 12085320

[19] Liu GY, Doran KS, Lawrence T, Turkson N, Puliti M, Tissi L, Nizet V. 2004. Sword and shield: linked group B streptococcal beta-hemolysin/cytolysin and carotenoid pigment function to subvert host phagocyte defense. Proc Natl Acad Sci U S A 101:14491–14496. doi:10.1073/pnas.0406143101 15381763 PMC521972

[20] Hensler ME, Liu GY, Sobczak S, Benirschke K, Nizet V, Heldt GP. 2005. Virulence role of group B Streptococcus beta-hemolysin/cytolysin in a neonatal rabbit model of early-onset pulmonary infection. J Infect Dis 191:1287–1291. doi:10.1086/428946 15776375

[21] Cieslewicz MJ, Chaffin D, Glusman G, Kasper D, Madan A, Rodrigues S, Fahey J, Wessels MR, Rubens CE. 2005. Structural and genetic diversity of group B streptococcus capsular polysaccharides. Infect Immun 73:3096–3103. doi:10.1128/IAI.73.5.3096-3103.2005 15845517 PMC1087335

[22] Noble K, Lu J, Guevara MA, Doster RS, Chambers SA, Rogers LM, Moore RE, Spicer SK, Eastman AJ, Francis JD, Manning SD, Rajagopal L, Aronoff DM, Townsend SD, Gaddy JA. 2021. Group B Streptococcus cpsE is required for serotype V capsule production and aids in biofilm formation and ascending infection of the reproductive tract during pregnancy. ACS Infect Dis 7:2686–2696. doi:10.1021/acsinfecdis.1c00182 34076405 PMC8588567

[23] Schuchat A. 1998. Epidemiology of group B streptococcal disease in the United States: shifting paradigms. Clin Microbiol Rev 11:497–513. doi:10.1128/CMR.11.3.497 9665980 PMC88893

[24] Madrid L, Seale AC, Kohli-Lynch M, Edmond KM, Lawn JE, Heath PT, Madhi SA, Baker CJ, Bartlett L, Cutland C, Gravett MG, Ip M, Le Doare K, Rubens CE, Saha SK, Sobanjo-Ter Meulen A, Vekemans J, Schrag S, Infant GBS Disease Investigator Group. 2017. Infant Group B streptococcal disease incidence and serotypes worldwide: systematic review and meta-analyses. Clin Infect Dis 65:S160–S172. doi:10.1093/cid/cix656 29117326 PMC5850457

[25] Jennings HJ, Lugowski C, Kasper DL. 1981. Conformational aspects critical to the immunospecificity of the type III group B streptococcal polysaccharide. Biochemistry 20:4511–4518. doi:10.1021/bi00519a001 6170311

[26] Edwards MS, Kasper DL, Jennings HJ, Baker CJ, Nicholson-Weller A. 1982. Capsular sialic acid prevents activation of the alternative complement pathway by type III, group B streptococci. J Immunol 128:1278–1283. 7035562

[27] Häyrinen J, Pelkonen S, Finne J. 1989. Structural similarity of the type-specific group B streptococcal polysaccharides and the carbohydrate units of tissue glycoproteins: evaluation of possible cross-reactivity. Vaccine (Auckl) 7:217–224. doi:10.1016/0264-410x(89)90232-6 2675485

[28] Morgenstern J, Campos Campos M, Nawroth P, Fleming T. 2020. The glyoxalase system-new insights into an ancient metabolism. Antioxidants (Basel) 9:939. doi:10.3390/antiox9100939 33019494 PMC7600140

[29] MacLean MJ, Ness LS, Ferguson GP, Booth IR. 1998. The role of glyoxalase I in the detoxification of methylglyoxal and in the activation of the KefB K+ efflux system in Escherichia coli. Mol Microbiol 27:563–571. doi:10.1046/j.1365-2958.1998.00701.x 9489668

[30] Lai SWT, Lopez Gonzalez EDJ, Zoukari T, Ki P, Shuck SC. 2022. Methylglyoxal and its adducts: induction, repair, and association with disease. Chem Res Toxicol 35:1720–1746. doi:10.1021/acs.chemrestox.2c00160 36197742 PMC9580021

[31] Spencer BL, Chatterjee A, Duerkop BA, Baker CJ, Doran KS. 2021. Complete genome sequence of neonatal clinical group B streptococcal isolate CJB111. Microbiol Resour Announc 10:e01268-20. doi:10.1128/MRA.01268-20 33446593 PMC7849706

[32] Burcham LR, Akbari MS, Alhajjar N, Keogh RA, Radin JN, Kehl-Fie TE, Belew AT, El-Sayed NM, McIver KS, Doran KS. 2022. Genomic analyses identify manganese homeostasis as a driver of group B streptococcal vaginal colonization. MBio 13:e0098522. doi:10.1128/mbio.00985-22 35658538 PMC9239048

[33] Burcham LR, Le Breton Y, Radin JN, Spencer BL, Deng L, Hiron A, Ransom MR, Mendonça J da C, Belew AT, El-Sayed NM, McIver KS, Kehl-Fie TE, Doran KS. 2020. Identification of zinc-dependent mechanisms used by group B Streptococcus to overcome calprotectin-mediated stress. MBio 11:e02302-20. doi:10.1128/mBio.02302-20 33173000 PMC7667036

[34] Inoue Y, Kimura A. 1995. Methylglyoxal and regulation of its metabolism in microorganisms. Adv Microb Physiol 37:177–227. doi:10.1016/s0065-2911(08)60146-0 8540421

[35] Poyart C, Pellegrini E, Marceau M, Baptista M, Jaubert F, Lamy M-C, Trieu-Cuot P. 2003. Attenuated virulence of Streptococcus agalactiae deficient in D-alanyl-lipoteichoic acid is due to an increased susceptibility to defensins and phagocytic cells. Mol Microbiol 49:1615–1625. doi:10.1046/j.1365-2958.2003.03655.x 12950925

[36] Cheng Q, Stafslien D, Purushothaman SS, Cleary P. 2002. The group B streptococcal C5a peptidase is both a specific protease and an invasin. Infect Immun 70:2408–2413. doi:10.1128/IAI.70.5.2408-2413.2002 11953377 PMC127948

[37] Dramsi S, Caliot E, Bonne I, Guadagnini S, Prévost M-C, Kojadinovic M, Lalioui L, Poyart C, Trieu-Cuot P. 2006. Assembly and role of pili in group B streptococci. Mol Microbiol 60:1401–1413. doi:10.1111/j.1365-2958.2006.05190.x 16796677

[38] Doran KS, Engelson EJ, Khosravi A, Maisey HC, Fedtke I, Equils O, Michelsen KS, Arditi M, Peschel A, Nizet V. 2005. Blood-brain barrier invasion by group B Streptococcus depends upon proper cell-surface anchoring of lipoteichoic acid. J Clin Invest 115:2499–2507. doi:10.1172/JCI23829 16138192 PMC1193870

[39] Whidbey C, Harrell MI, Burnside K, Ngo L, Becraft AK, Iyer LM, Aravind L, Hitti J, Adams Waldorf KM, Rajagopal L. 2013. A hemolytic pigment of Group B Streptococcus allows bacterial penetration of human placenta. J Exp Med 210:1265–1281. doi:10.1084/jem.20122753 23712433 PMC3674703

[40] Boldenow E, Gendrin C, Ngo L, Bierle C, Vornhagen J, Coleman M, Merillat S, Armistead B, Whidbey C, Alishetti V, Santana-Ufret V, Ogle J, Gough M, Srinouanprachanh S, MacDonald JW, Bammler TK, Bansal A, Liggitt HD, Rajagopal L, Adams Waldorf KM. 2016. Group B Streptococcus circumvents neutrophils and neutrophil extracellular traps during amniotic cavity invasion and preterm labor. Sci Immunol 1:eaah4576. doi:10.1126/sciimmunol.aah4576 27819066 PMC5089172

[41] Papasergi S, Brega S, Mistou M-Y, Firon A, Oxaran V, Dover R, Teti G, Shai Y, Trieu-Cuot P, Dramsi S. 2011. The GBS PI-2a pilus is required for virulence in mice neonates. PLoS One 6:e18747. doi:10.1371/journal.pone.0018747 21525979 PMC3078112

[42] Patras KA, Derieux J, Al-Bassam MM, Adiletta N, Vrbanac A, Lapek JD, Zengler K, Gonzalez DJ, Nizet V. 2018. Group B Streptococcus biofilm regulatory protein a contributes to bacterial physiology and innate immune resistance. J Infect Dis 218:1641–1652. doi:10.1093/infdis/jiy341 29868829 PMC6173572

[43] Mercado-Evans V, Mejia ME, Zulk JJ, Ottinger S, Hameed ZA, Serchejian C, Marunde MG, Robertson CM, Ballard MB, Ruano SH, Korotkova N, Flores AR, Pennington KA, Patras KA. 2024. Gestational diabetes augments group B Streptococcus infection by disrupting maternal immunity and the vaginal microbiota. Nat Commun 15:1035. doi:10.1038/s41467-024-45336-6 38310089 PMC10838280

[44] Carlin AF, Chang Y-C, Areschoug T, Lindahl G, Hurtado-Ziola N, King CC, Varki A, Nizet V. 2009. Group B Streptococcus suppression of phagocyte functions by protein-mediated engagement of human Siglec-5. J Exp Med 206:1691–1699. doi:10.1084/jem.20090691 19596804 PMC2722167

[45] Uchiyama S, Sun J, Fukahori K, Ando N, Wu M, Schwarz F, Siddiqui SS, Varki A, Marth JD, Nizet V. 2019. Dual actions of group B Streptococcus capsular sialic acid provide resistance to platelet-mediated antimicrobial killing. Proc Natl Acad Sci U S A 116:7465–7470. doi:10.1073/pnas.1815572116 30910970 PMC6462088

[46] Moulin P, Patron K, Cano C, Zorgani MA, Camiade E, Borezée-Durant E, Rosenau A, Mereghetti L, Hiron A. 2016. The Adc/Lmb system mediates zinc acquisition in Streptococcus agalactiae and contributes to bacterial growth and survival. J Bacteriol 198:3265–3277. doi:10.1128/JB.00614-16 27672194 PMC5116930

[47] Akbari MS, Keogh RA, Radin JN, Sanchez-Rosario Y, Johnson MDL, Horswill AR, Kehl-Fie TE, Burcham LR, Doran KS. 2023. The impact of nutritional immunity on Group B streptococcal pathogenesis during wound infection. MBio 14:e0030423. doi:10.1128/mbio.00304-23 37358277 PMC10470527

[48] Clancy A, Loar JW, Speziali CD, Oberg M, Heinrichs DE, Rubens CE. 2006. Evidence for siderophore-dependent iron acquisition in group B streptococcus. Mol Microbiol 59:707–721. doi:10.1111/j.1365-2958.2005.04974.x 16390461

[49] Shabayek S, Bauer R, Mauerer S, Mizaikoff B, Spellerberg B. 2016. A streptococcal NRAMP homologue is crucial for the survival of Streptococcus agalactiae under low pH conditions. Mol Microbiol 100:589–606. doi:10.1111/mmi.13335 27150893

[50] Sullivan MJ, Goh KGK, Gosling D, Katupitiya L, Ulett GC. 2021. Copper intoxication in group B Streptococcus triggers transcriptional activation of the cop operon that contributes to enhanced virulence during acute infection. J Bacteriol 203:e0031521. doi:10.1128/JB.00315-21 34251869 PMC8447484

[51] Faralla C, Metruccio MM, De Chiara M, Mu R, Patras KA, Muzzi A, Grandi G, Margarit I, Doran KS, Janulczyk R. 2014. Analysis of two-component systems in group B Streptococcus shows that RgfAC and the novel FspSR modulate virulence and bacterial fitness. MBio 5:e00870–14. doi:10.1128/mBio.00870-14 24846378 PMC4030450

[52] Thomas L, Cook L. 2020. Two-component signal transduction systems in the human pathogen Streptococcus agalactiae. Infect Immun 88:e00931-19. doi:10.1128/IAI.00931-19 31988177 PMC7309623

[53] Ipe DS, Ben Zakour NL, Sullivan MJ, Beatson SA, Ulett KB, Benjamin WH Jr, Davies MR, Dando SJ, King NP, Cripps AW, Schembri MA, Dougan G, Ulett GC. 2016. Discovery and characterization of human-urine utilization by asymptomatic-bacteriuria-causing Streptococcus agalactiae. Infect Immun 84:307–319. doi:10.1128/IAI.00938-15 26553467 PMC4694007

[54] Poyart C, Lamy MC, Boumaila C, Fiedler F, Trieu-Cuot P. 2001. Regulation of D-alanyl-lipoteichoic acid biosynthesis in Streptococcus agalactiae involves a novel two-component regulatory system. J Bacteriol 183:6324–6334. doi:10.1128/JB.183.21.6324-6334.2001 11591677 PMC100127

[55] Quach D, van Sorge NM, Kristian SA, Bryan JD, Shelver DW, Doran KS. 2009. The CiaR response regulator in group B Streptococcus promotes intracellular survival and resistance to innate immune defenses. J Bacteriol 191:2023–2032. doi:10.1128/JB.01216-08 19114476 PMC2655536

[56] Yang Y, Luo M, Zhou H, Li C, Luk A, Zhao G, Fung K, Ip M. 2019. Role of two-component system response regulator bceR in the antimicrobial resistance, virulence, biofilm formation, and stress response of Group B Streptococcus. Front Microbiol 10. doi:10.3389/fmicb.2019.00010 PMC6351488 30728810

[57] Khosa S, AlKhatib Z, Smits SHJ. 2013. NSR from Streptococcus agalactiae confers resistance against nisin and is encoded by a conserved NSR operon. Biol Chem 394:1543–1549. doi:10.1515/hsz-2013-0167 23893686

[58] Khosa S, Frieg B, Mulnaes D, Kleinschrodt D, Hoeppner A, Gohlke H, Smits SHJ. 2016. Structural basis of lantibiotic recognition by the nisin resistance protein from Streptococcus agalactiae. Sci Rep 6:18679. doi:10.1038/srep18679 26727488 PMC4698656

[59] Rajagopal L, Vo A, Silvestroni A, Rubens CE. 2005. Regulation of purine biosynthesis by a eukaryotic-type kinase in Streptococcus agalactiae. Mol Microbiol 56:1329–1346. doi:10.1111/j.1365-2958.2005.04620.x 15882424 PMC2366208

[60] Tamura GS, Nittayajarn A, Schoentag DL. 2002. A glutamine transport gene, glnQ, is required for fibronectin adherence and virulence of group B streptococci. Infect Immun 70:2877–2885. doi:10.1128/IAI.70.6.2877-2885.2002 12010975 PMC128016

[61] Ipe DS, Sullivan MJ, Goh KGK, Hashimi SM, Munn AL, Ulett GC. 2021. Conserved bacterial de novo guanine biosynthesis pathway enables microbial survival and colonization in the environmental niche of the urinary tract. ISME J 15:2158–2162. doi:10.1038/s41396-021-00934-w 33649549 PMC8245529

[62] Santi I, Grifantini R, Jiang S-M, Brettoni C, Grandi G, Wessels MR, Soriani M. 2009. CsrRS regulates group B Streptococcus virulence gene expression in response to environmental pH: a new perspective on vaccine development. J Bacteriol 191:5387–5397. doi:10.1128/JB.00370-09 19542277 PMC2725634

[63] Yang Q, Zhang M, Harrington DJ, Black GW, Sutcliffe IC. 2011. A proteomic investigation of Streptococcus agalactiae reveals that human serum induces the C protein β antigen and arginine deiminase. Microbes Infect 13:757–760. doi:10.1016/j.micinf.2011.03.001 21458589

[64] van de Rijn I, Kessler RE. 1980. Growth characteristics of group A streptococci in A new chemically defined medium. Infect Immun 27:444–448. doi:10.1128/iai.27.2.444-448.1980 6991416 PMC550785

[65] Zhang MM, Ong CY, Walker MJ, McEwan AG. 2016. Defence against methylglyoxal in group A Streptococcus: a role for glyoxylase I in bacterial virulence and survival in neutrophils? Pathog Dis 74:ftv122. doi:10.1093/femspd/ftv122 26702634

[66] Anaya-Sanchez A, Feng Y, Berude JC, Portnoy DA. 2021. Detoxification of methylglyoxal by the glyoxalase system is required for glutathione availability and virulence activation in Listeria monocytogenes. PLoS Pathog 17:e1009819. doi:10.1371/journal.ppat.1009819 34407151 PMC8372916

[67] He MM, Clugston SL, Honek JF, Matthews BW. 2000. Determination of the structure of Escherichia coli glyoxalase I suggests a structural basis for differential metal activation. Biochemistry 39:8719–8727. doi:10.1021/bi000856g 10913283

[68] O’Neill LAJ, Kishton RJ, Rathmell J. 2016. A guide to immunometabolism for immunologists. Nat Rev Immunol 16:553–565. doi:10.1038/nri.2016.70 27396447 PMC5001910

[69] Darwin KH, Stanley SA. 2022. The aldehyde hypothesis: metabolic intermediates as antimicrobial effectors. Open Biol 12:220010. doi:10.1098/rsob.220010 35414258 PMC9006002

[70] Limón G, Samhadaneh NM, Pironti A, Darwin KH. 2023. Aldehyde accumulation in Mycobacterium tuberculosis with defective proteasomal degradation results in copper sensitivity. MBio 14:e0036323. doi:10.1128/mbio.00363-23 37350636 PMC10470581

[71] Rachman H, Kim N, Ulrichs T, Baumann S, Pradl L, Nasser Eddine A, Bild M, Rother M, Kuban R-J, Lee JS, Hurwitz R, Brinkmann V, Kosmiadi GA, Kaufmann SHE. 2006. Critical role of methylglyoxal and AGE in mycobacteria-induced macrophage apoptosis and activation. PLoS One 1:e29. doi:10.1371/journal.pone.0000029 17183656 PMC1762319

[72] Jeon JH, Hong CW, Kim EY, Lee JM. 2020. Current understanding on the metabolism of neutrophils. Immune Netw 20:e46. doi:10.4110/in.2020.20.e46 33425431 PMC7779868

[73] Daley JM, Thomay AA, Connolly MD, Reichner JS, Albina JE. 2008. Use of Ly6G-specific monoclonal antibody to deplete neutrophils in mice. J Leukoc Biol 83:64–70. doi:10.1189/jlb.0407247 17884993

[74] Goldenberg RL, Hauth JC, Andrews WW. 2000. Intrauterine infection and preterm delivery. N Engl J Med 342:1500–1507. doi:10.1056/NEJM200005183422007 10816189

[75] Hooven TA, Catomeris AJ, Bonakdar M, Tallon LJ, Santana-Cruz I, Ott S, Daugherty SC, Tettelin H, Ratner AJ. 2018. The streptococcus agalactiae stringent response enhances virulence and persistence in human blood. Infect Immun 86:e00612-17. doi:10.1128/IAI.00612-17 29109175 PMC5736797

[76] Mereghetti L, Sitkiewicz I, Green NM, Musser JM. 2009. Identification of an unusual pattern of global gene expression in group B Streptococcus grown in human blood. PLoS ONE 4:e7145. doi:10.1371/journal.pone.0007145 19774088 PMC2745576

[77] Di Palo B, Rippa V, Santi I, Brettoni C, Muzzi A, Metruccio MME, Grifantini R, Telford JL, Paccani SR, Soriani M. 2013. Adaptive response of Group B streptococcus to high glucose conditions: new insights on the CovRS regulation network. PLoS One 8:e61294. doi:10.1371/journal.pone.0061294 23585887 PMC3621830

[78] López Y, Parra E, Cepas V, Sanfeliú I, Juncosa T, Andreu A, Xercavins M, Pérez J, Sanz S, Vergara A, Bosch J, Soto SM. 2018. Serotype, virulence profile, antimicrobial resistance and macrolide-resistance determinants in Streptococcus agalactiae isolates in pregnant women and neonates in Catalonia, Spain. Enfermedades Infecciosas y Microbiología Clínica 36:472–477. doi:10.1016/j.eimc.2017.08.006 29029763

[79] Zhang X, Schalkwijk CG, Wouters K. 2022. Immunometabolism and the modulation of immune responses and host defense: a role for methylglyoxal? Biochim Biophys Acta Mol Basis Dis 1868:166425. doi:10.1016/j.bbadis.2022.166425 35500827

[80] Beisswenger PJ. 2014. Methylglyoxal in diabetes: link to treatment, glycaemic control and biomarkers of complications. Biochem Soc Trans 42:450–456. doi:10.1042/BST20130275 24646259

[81] Lee C, Park C. 2017. Bacterial responses to glyoxal and methylglyoxal: reactive electrophilic species. Int J Mol Sci 18:169. doi:10.3390/ijms18010169 28106725 PMC5297802

[82] Hazen SL, Hsu FF, d’Avignon A, Heinecke JW. 1998. Human neutrophils employ myeloperoxidase to convert alpha-amino acids to a battery of reactive aldehydes: a pathway for aldehyde generation at sites of inflammation. Biochemistry 37:6864–6873. doi:10.1021/bi972449j 9578573

[83] Furuta A, Brokaw A, Manuel G, Dacanay M, Marcell L, Seepersaud R, Rajagopal L, Adams Waldorf K. 2022. Bacterial and host determinants of group B streptococcal infection of the neonate and infant. Front Microbiol 13:820365. doi:10.3389/fmicb.2022.820365 35265059 PMC8899651

[84] Clugston SL, Yajima R, Honek JF. 2004. Investigation of metal binding and activation of Escherichia coli glyoxalase I: kinetic, thermodynamic and mutagenesis studies. Biochem J 377:309–316. doi:10.1042/BJ20030271 14556652 PMC1223881

[85] Suttisansanee U, Lau K, Lagishetty S, Rao KN, Swaminathan S, Sauder JM, Burley SK, Honek JF. 2011. Structural variation in bacterial glyoxalase I enzymes: investigation of the metalloenzyme glyoxalase I from Clostridium acetobutylicum. J Biol Chem 286:38367–38374. doi:10.1074/jbc.M111.251603 21914803 PMC3207458

[86] Zeng L, Noeparvar P, Burne RA, Glezer BS. 2024. Genetic characterization of glyoxalase pathway in oral streptococci and its contribution to interbacterial competition. J Oral Microbiol 16:2322241. doi:10.1080/20002297.2024.2322241 38440286 PMC10911100

[87] Cook LCC, Hu H, Maienschein-Cline M, Federle MJ. 2018. A vaginal tract signal detected by the group B Streptococcus SaeRS system elicits transcriptomic changes and enhances murine colonization. Infect Immun 86:e00762-17. doi:10.1128/IAI.00762-17 29378799 PMC5865029

[88] Alfano M, Cavazza C. 2020. Structure, function, and biosynthesis of nickel-dependent enzymes. Protein Sci 29:1071–1089. doi:10.1002/pro.3836 32022353 PMC7184782

[89] Eitinger T, Mandrand-Berthelot MA. 2000. Nickel transport systems in microorganisms. Arch Microbiol 173:1–9. doi:10.1007/s002030050001 10648098

[90] Krymkiewicz N, Diéguez E, Rekarte UD, Zwaig N. 1971. Properties and mode of action of a bactericidal compound (=methylglyoxal) produced by a mutant of Escherichia coli. J Bacteriol 108:1338–1347. doi:10.1128/jb.108.3.1338-1347.1971 4945198 PMC247224

[91] Takahashi K. 1977. Further studies on the reactions of phenylglyoxal and related reagents with proteins. J Biochem 81:403–414. doi:10.1093/oxfordjournals.jbchem.a131472 321441

[92] Takahashi K. 1977. The reactions of phenylglyoxal and related reagents with amino acids. J Biochem 81:395–402. doi:10.1093/oxfordjournals.jbchem.a131471 14946

[93] Cheung ST, Fonda ML. 1979. Kinetics of the inactivation of Escherichia coli glutamate apodecarboxylase by phenylglyoxal. Arch Biochem Biophys 198:541–547. doi:10.1016/0003-9861(79)90529-0 42358

[94] Rabbani N, Thornalley PJ. 2014. Measurement of methylglyoxal by stable isotopic dilution analysis LC-MS/MS with corroborative prediction in physiological samples. Nat Protoc 9:1969–1979. doi:10.1038/nprot.2014.129 25058644

[95] Schalkwijk CG, Stehouwer CDA. 2020. Methylglyoxal, a highly reactive dicarbonyl compound, in diabetes, its vascular complications, and other age-related diseases. Physiol Rev 100:407–461. doi:10.1152/physrev.00001.2019 31539311

[96] Keogh RA, Haeberle AL, Langouët-Astrié CJ, Kavanaugh JS, Schmidt EP, Moore GD, Horswill AR, Doran KS. 2022. Group B Streptococcus adaptation promotes survival in a hyperinflammatory diabetic wound environment. Sci Adv 8:eadd3221. doi:10.1126/sciadv.add3221 36367946 PMC9651866

[97] Kant S, Liu L, Vazquez-Torres A. 2023. The methylglyoxal pathway is a sink for glutathione in Salmonella experiencing oxidative stress. PLoS Pathog 19:e1011441. doi:10.1371/journal.ppat.1011441 37267419 PMC10266609

[98] Joyce LR, Manzer HS, da C Mendonça J, Villarreal R, Nagao PE, Doran KS, Palmer KL, Guan Z. 2022. Identification of a novel cationic glycolipid in Streptococcus agalactiae that contributes to brain entry and meningitis. PLoS Biol 20:e3001555. doi:10.1371/journal.pbio.3001555 35180210 PMC8893666

[99] Banerjee A, Kim BJ, Carmona EM, Cutting AS, Gurney MA, Carlos C, Feuer R, Prasadarao NV, Doran KS. 2011. Bacterial Pili exploit integrin machinery to promote immune activation and efficient blood-brain barrier penetration. Nat Commun 2:462. doi:10.1038/ncomms1474 21897373 PMC3195231

[100] Kim BJ, Hancock BM, Bermudez A, Del Cid N, Reyes E, van Sorge NM, Lauth X, Smurthwaite CA, Hilton BJ, Stotland A, Banerjee A, Buchanan J, Wolkowicz R, Traver D, Doran KS. 2015. Bacterial induction of Snail1 contributes to blood-brain barrier disruption. J Clin Invest 125:2473–2483. doi:10.1172/JCI74159 25961453 PMC4497739

[101] Spencer BL, Deng L, Patras KA, Burcham ZM, Sanches GF, Nagao PE, Doran KS. 1930. Cas9 contributes to group B streptococcal colonization and disease. Front Microbiol 10. doi:10.3389/fmicb.2019.01930 PMC6712506 31497003

[102] Wingett SW, Andrews S. 2018. FastQ screen: a tool for multi-genome mapping and quality control. F1000Res 7:1338. doi:10.12688/f1000research.15931.2 30254741 PMC6124377

[103] Martin M. 2011. Cutadapt removes adapter sequences from high-throughput sequencing reads. EMBnet J 17:10. doi:10.14806/ej.17.1.200

[104] DeJesus MA, Ambadipudi C, Baker R, Sassetti C, Ioerger TR. 2015. TRANSIT--a software tool for Himar1 TnSeq analysis. PLoS Comput Biol 11:e1004401. doi:10.1371/journal.pcbi.1004401 26447887 PMC4598096

[105] Li H, Durbin R. 2009. Fast and accurate short read alignment with Burrows-Wheeler transform. Bioinformatics 25:1754–1760. doi:10.1093/bioinformatics/btp324 19451168 PMC2705234

[106] Madeira F, Pearce M, Tivey ARN, Basutkar P, Lee J, Edbali O, Madhusoodanan N, Kolesnikov A, Lopez R. 2022. Search and sequence analysis tools services from EMBL-EBI in 2022. Nucleic Acids Res 50:W276–W279. doi:10.1093/nar/gkac240 35412617 PMC9252731

[107] Robert X, Gouet P. 2014. Deciphering key features in protein structures with the new ENDscript server. Nucleic Acids Res 42:W320–4. doi:10.1093/nar/gku316 24753421 PMC4086106

[108] Altschul SF, Madden TL, Schäffer AA, Zhang J, Zhang Z, Miller W, Lipman DJ. 1997. Gapped BLAST and PSI-BLAST: a new generation of protein database search programs. Nucleic Acids Res 25:3389–3402. doi:10.1093/nar/25.17.3389 9254694 PMC146917

[109] Altschul SF, Wootton JC, Gertz EM, Agarwala R, Morgulis A, Schäffer AA, Yu Y-K. 2005. Protein database searches using compositionally adjusted substitution matrices. FEBS J 272:5101–5109. doi:10.1111/j.1742-4658.2005.04945.x 16218944 PMC1343503

[110] Jumper J, Evans R, Pritzel A, Green T, Figurnov M, Ronneberger O, Tunyasuvunakool K, Bates R, Žídek A, Potapenko A, et al.. 2021. Highly accurate protein structure prediction with AlphaFold. Nature New Biol 596:583–589. doi:10.1038/s41586-021-03819-2 PMC8371605 34265844

[111] Mirdita M, Schütze K, Moriwaki Y, Heo L, Ovchinnikov S, Steinegger M. 2022. ColabFold: making protein folding accessible to all. Nat Methods 19:679–682. doi:10.1038/s41592-022-01488-1 35637307 PMC9184281

[112] Berman HM, Westbrook J, Feng Z, Gilliland G, Bhat TN, Weissig H, Shindyalov IN, Bourne PE. 2000. The protein data bank. Nucleic Acids Res 28:235–242. doi:10.1093/nar/28.1.235 10592235 PMC102472

[113] Joyce LR, Kim S, Spencer BL, Christensen PM, Palmer KL, Guan Z, Siegenthaler JA, Doran KS. 2024. Streptococcus agalactiae glycolipids promote virulence by thwarting immune cell clearance. Sci Adv 10:eadn7848. doi:10.1126/sciadv.adn7848 38809989 PMC11135403

