# Supplementary figures and images for: Identification of glyoxalase A in group B Streptococcus and its contribution to methylglyoxal tolerance and virulence
Source: Infect Immun. 2025 Feb 26;93(4):e00540-24. doi: 10.1128/iai.00540-24 (PMC11977320; doi:10.1128/iai.00540-24)

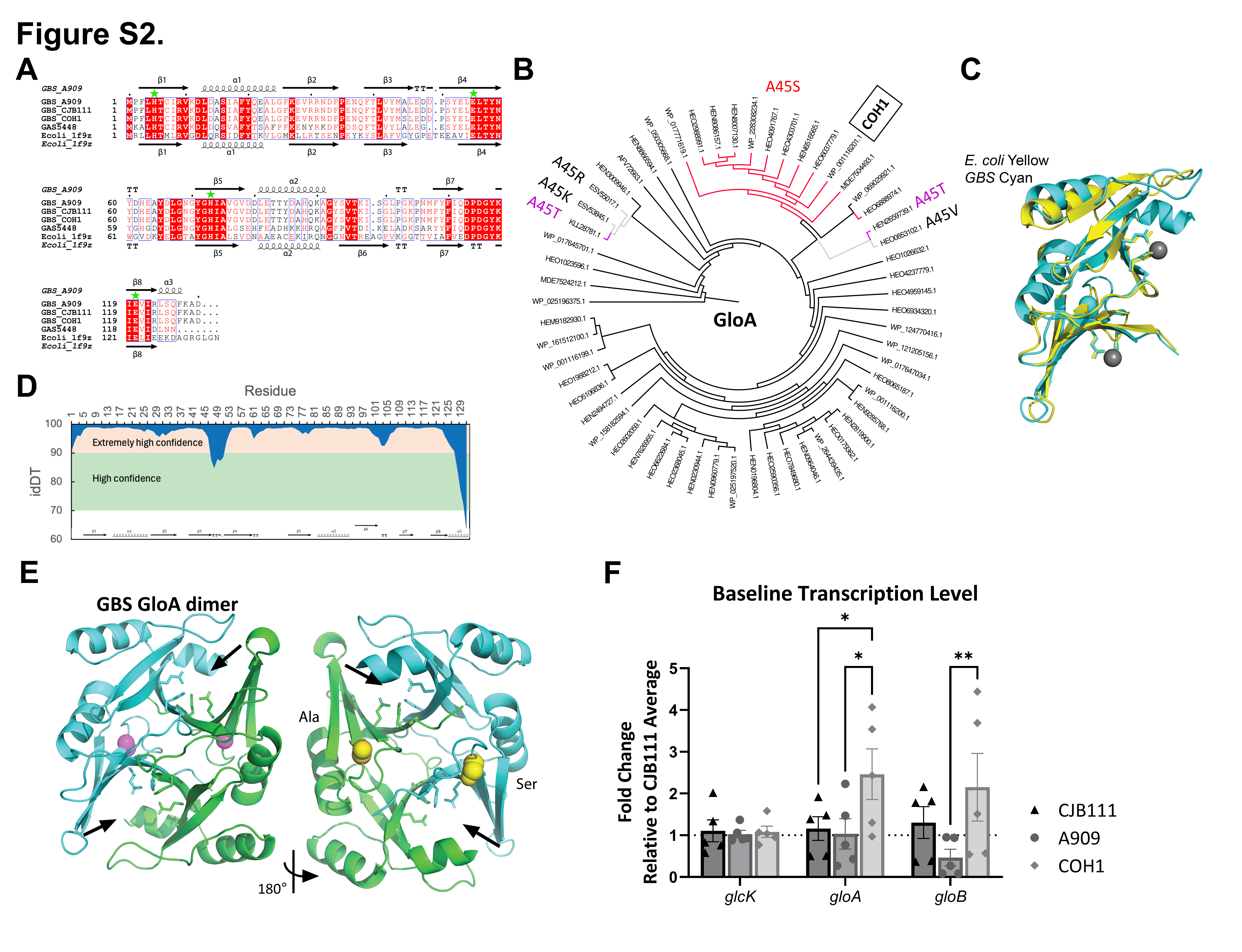

Supplement: Fig. S2 — GloA protein characterization in GBS. [file iai.00540-24-s0002.tif]

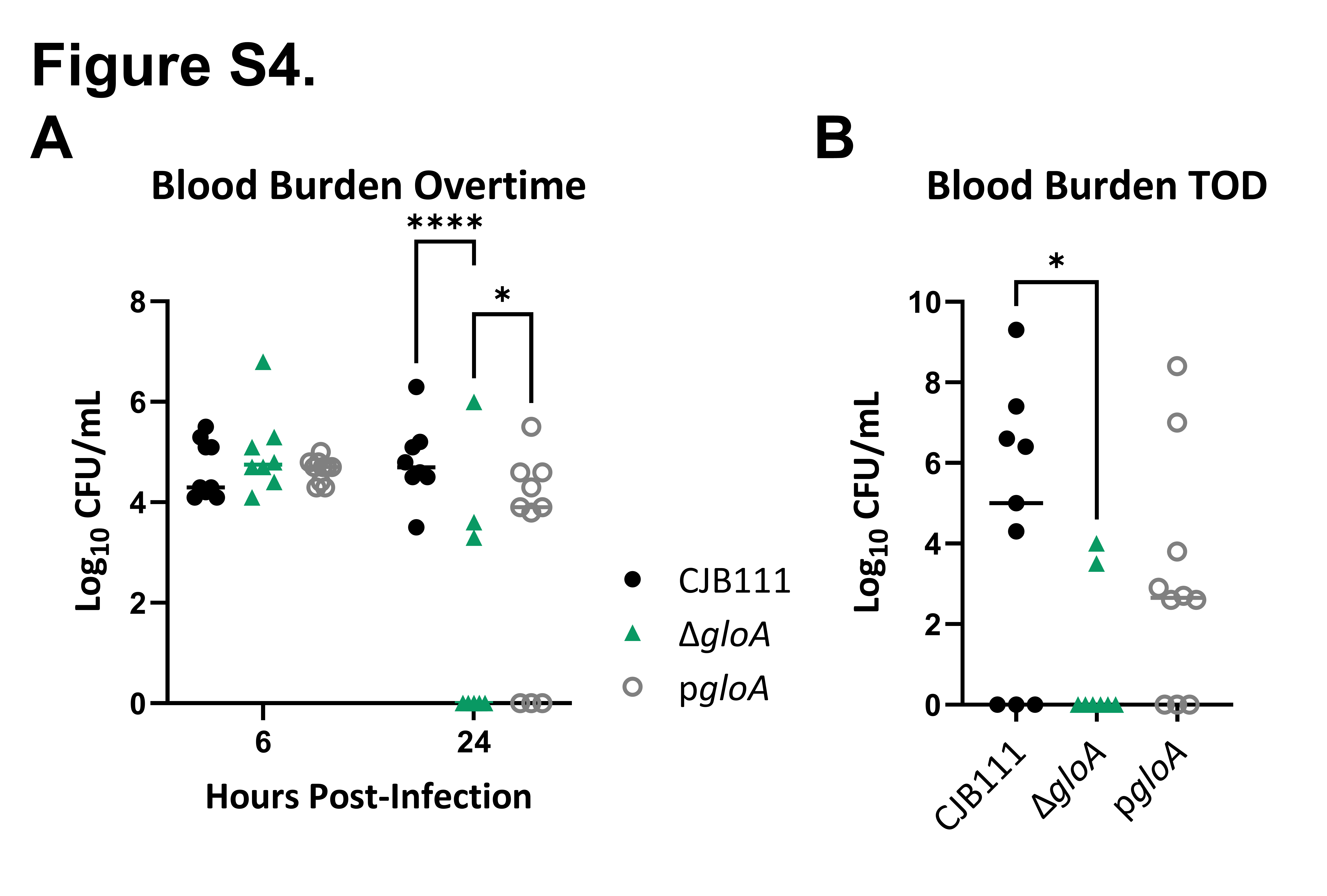

Supplement: Fig. S4 — In vivo infection with complemented strain and early blood timepoint. [file iai.00540-24-s0004.tif]
