# Supplementary material for: Identification of glyoxalase A in group B Streptococcus and its contribution to methylglyoxal tolerance and virulence
Source: Infect Immun. 2025 Feb 26;93(4):e00540-24. doi: 10.1128/iai.00540-24 (PMC11977320; doi:10.1128/iai.00540-24)
Supplement: Supplemental material — Legends for supplemental figures and tables. [file iai.00540-24-s0006.docx]

**Supplementary Material**

**Table S1.** Complete *in vivo* blood Tn-sequencing dataset.

**Table S2.** Primers and Strains

**Figure S1**. The impact of MG on different GBS isolates. Growth curves for (A) CJB111 in mCDM with 0.5 mM and 1.0 mM MG quantified by CFU enumeration or (B) Growth curves for 10 GBS isolates in mCDM without MG (left) or with 1.0 mM MG (right) quantified by OD_600_.

**Figure S2**. GBS Glyoxalase A protein characterization. (A) Alignment of GloA amino acid sequences from GBS CJB111, GBS A909, GBS COH1, *S. pyogenes* 5448, and *E. coli* K-12. Green stars indicate known or predicted metal binding sites and colored bar indicates confidence of structure prediction. (B) Phylogenetic tree for 57 GBS GloA proteins. Proteins/branches with a mutation in amino acid residue A45 are labeled and colored. Red indicates an A45S mutation, purple indicates an A45T mutation, and grey indicates a mutation that only occurred once. (C) Superimposed tertiary structures for the solved *E. coli* GloA and the predicted GBS GloA. (D) idDT confidence scores across GBS GloA predicted structure. (E) Left: Predicted tertiary protein dimer for GBS A909/CJB111 GloA. Right: 180° horizontal rotation of the predicted tertiary protein dimer with the blue monomer containing GBS COH1 A45S mutation. Black arrows indicate predicted active site. (F) Baseline transcription of *gloA* and *gloB* genes grown to mid-log in mCDM and quantified by RT-qPCR. Gene transcript levels were normalized to the average CJB111 levels. Significance determined by 2way ANOVA with uncorrected Fisher’s LSD test, *P* < 0.05. * < 0.05, ** < 0.01.

**Figure S3.** The impact of MG and Glyoxalase A on GBS growth and virulence. (A) Growth curves for CJB111 in mCDM with or without MG after pre-exposure to 0.5 mM MG quantified by OD_600_. Growth curves for CJB111, ∆*gloA*, and p*gloA* strains in mCDM (B) with 10, 22, or 50 mM glucose quantified by OD_600_ or (C) with 0.1% (29.4mM) hydrogen peroxide quantified by CFU enumeration. (D) Percent red blood cell lysis of human blood for CJB111, Δ*gloA*, and p*gloA* strains relative to positive lysis control at 24hrs post-inoculation.

**Figure S4.** MG detoxification is more important as infection progresses. Recovered CFU counts from the blood of infected mice at (A) 6 and 24 hours post-infection and (B) TOD. Significance determined by (A) Mixed-effects analysis with uncorrected Fisher’s LSD test, or (B) Kruskal-Wallis with uncorrected Dunn’s test. , *P* < 0.05. * < 0.05, ****<0.0001.

**Figure S5.** The impact of serum, primary cells, and glucose on neutrophil phenotypes. (A) Survival of CJB111, Δ*gloA*, and p*gloA* strains over time in the presence of serum without HL60-neutrophils. Percent survival was calculated by dividing CFU recovered from wells with GBS opsonized with normal serum by CFU recovered from wells with GBS opsonized with heat-killed (HK) serum. (B) Cytotoxicity of HL60-neutrophils incubated with CJB111, ∆*gloA*, and p*gloA* that had been opsonized with HK or normal serum after 5 hrs of infection. (C) Recovered CFU counts normalized to the WT average of WT CJB111, ∆*gloA*, and p*gloA* strains after 4 hrs of infection of BMNs. (D) Flow cytometry quantification of intracellular MG-modified proteins in HL60-neutrophils with or without WT CJB111 infection in 0mM glucose media. Left: Representative histogram displaying MG signal that includes isotype control, uninfected, and infected cells. Right: Geometric MFI quantification for MG in uninfected and infected samples. Significance determined by (C) one-way ANOVA with Uncorrected Fisher’s LSD test or (D) Unpaired Student *t* test, *P* < 0.05.
